# Dynamic Changes in Volatile Compounds of Shaken Black Tea during Its Manufacture by GC × GC–TOFMS and Multivariate Data Analysis

**DOI:** 10.3390/foods11091228

**Published:** 2022-04-25

**Authors:** Jinjin Xue, Panpan Liu, Junfeng Yin, Weiwei Wang, Jianyong Zhang, Wei Wang, Ting Le, Dejiang Ni, Heyuan Jiang

**Affiliations:** 1 Key Laboratory of Tea Biology and Resources Utilization, Ministry of Agriculture, Tea Research Institute, Chinese Academy of Agricultural Sciences, Hangzhou 310008, China; xuejinjin911@163.com (J.X.); yinjf@tricaas.com (J.Y.); wangwei11211@tricaas.com (W.W.); zjy5128@tricaas.com (J.Z.); ww1040491839@163.com (W.W.); letin@tricaas.com (T.L.); 2 Key Laboratory of Horticulture Plant Biology, Ministry of Education, College of Horticulture and Forestry Sciences, Huazhong Agricultural University, Wuhan 430070, China; 3 Fruit and Tea Research Institute, Hubei Academy of Agricultural Sciences, Wuhan 430064, China; liuppitea@163.com

**Keywords:** shaken black tea, volatile compounds, GC × GC–TOFMS, relative odor activity value, multivariate data analysis

## Abstract

Changes in key odorants of shaken black tea (SBT) during its manufacture were determined using headspace solid-phase microextraction (HS-SPME) combined with comprehensive two-dimensional gas chromatography–time-of-flight mass spectrometry (GC × GC–TOFMS) and multivariate data analysis. A total of 241 volatiles was identified, comprising 49 aldehydes, 40 esters, 29 alcohols, 34 ketones, 30 aromatics, 24 alkenes, 17 alkanes, 13 furans, and 5 other compounds. A total of 27 volatiles had average relative odor activity values (rOAVs) greater than 1, among which (E)-β-ionone, (E,Z)-2,6-nonadienal, and 1-octen-3-one exhibited the highest values. According to the criteria of variable importance in projection (VIP) > 1, *p* < 0.05, and |log_2_FC| > 1, 61 discriminatory volatile compounds were screened out, of which 26 substances were shared in the shaking stage (FL vs. S1, S1 vs. S2, S2 vs. S3). The results of the orthogonal partial least squares discriminate analysis (OPLS-DA) differentiated the influence of shaking, fermentation, and drying processes on the formation of volatile compounds in SBT. In particular, (Z)-3-hexenol, (Z)-hexanoic acid, 3-hexenyl ester, (E)-β-farnesene, and indole mainly formed in the shaking stage, which promoted the formation of the floral and fruity flavor of black tea. This study enriches the basic theory of black tea flavor quality and provide the theoretical basis for the further development of aroma quality control.

## 1. Introduction

Tea (*Camellia sinensis* L.) is one of the most widely consumed beverages in the world, with a satisfactory taste and rich aroma. Black tea (fully fermented) accounts for 75% of global tea consumption due to its unique flavor as well as its human health benefits [1]. The shape, soup color, aroma, taste, and leaf bottom of tea are the quality indicators, and therefore aroma profile is an important factor in determining its quality [2]. The distribution, relative proportions, and complex perceptual interactions of volatile compounds contribute to the overall presentation of tea aroma quality [3]. The aroma quality of tea is undeniably affected by the differences in volatile compounds, which are influenced by cultivar [4], cultivation conditions [5], elevation [6], processing methods [7], storage [8], and brewing conditions [9]. Among them, processing technology plays an undeniably importance role in the formation of the unique flavor profile of black tea [10].

Shaking is a key process in the processing of oolong tea, and it is also the basis and guarantee for the formation of its unique natural flower and fruit aroma [11]. The shaking process of oolong tea was introduced into the processing technology of traditional black tea to produce shaken black tea (SBT), the process of which includes withering, shaking, rolling, fermentation, and drying. In recent years, relevant research on the improvement of tea processing technology has shown that the introduction of the shaking process into traditional black tea can improve its aroma quality, which may be mainly due to the hydrolase (such as β-glucosidase) activity of glycoside aroma precursors in fresh leaves enhanced by the stimulation of stress (dehydration and mechanical damage) during shaking [12,13]. Compared with traditional black teas, SBT had a strong fragrance, fresh and mellow taste [14], and is deeply loved by consumers. Studies have shown that the main aroma substances of SBT are nerolidol, β-linalool, (Z)-hexanoic acid, 3-hexenyl ester, geraniol, etc. [15]. Shaking can significantly reduce alcohols and increase the aroma content of aldehydes, esters, acids, and ketones, thereby improving the aroma quality of tea [16].

Gas chromatography–mass spectrometry (GC–MS) has always been the mainstream method for the analysis of volatile components in tea [11,17]. Compared with traditional GC–MS, comprehensive two-dimensional gas chromatography–time-of-flight mass spectrometry (GC × GC–TOFMS) has the advantages of high sensitivity, high resolution, large peak capacity, qualitative accuracy, and a large amount of information [18]. In recent years, it has been widely used in the study of volatile components of tea [19,20]. Due to the small number of volatile components detected by GC–MS, the aroma composition of volatile substances cannot be fully reflected, and previous research has often focused on single processes of withering, rolling, fermentation, or drying. Therefore, GC × GC–TOFMS was used in this study to characterize the volatile components of SBT. The orthogonal partial least squares discriminate analysis (OPLS-DA) was used to carry out pairwise comparison analysis of samples in the processing stage, and the differential compounds were screened out to reveal the dynamic aroma changes of volatile organic compounds (VOCs) during the whole process and clarify the influence of processing on VOCs. This study will provide a comprehensive profile of volatile changes during SBT processing, which is potentially important for flavor quality improvement of black tea.

## 2. Materials and Methods

### 2.1. Tea Leaf Samples and Process Characterization

Tea leaf samples from different stages of the manufacturing process were obtained from the Tea Research Institute, Chinese Academy of Agricultural Sciences, in September 2020. The fresh leaves (FL) of the “Fudingdabai” variety with one bud with two leaves were placed on a bamboo sieve and dried in the sun for 20 min. Then the leaves were moved into the room and withered for 4 h (25 °C). The leaves subsequently underwent a shaking process: the leaves were turned over three times, shaking sieved by hand for 5 min, then spread and left for 1.5 h each time (S1–S3). Subsequently, indoor withering was carried out for 6 h, and the moisture of the leaves was approximately 60% for rolling. The rolling process followed the principle of light pressure first and then heavy pressure for a total of 60 min. Fermentation was carried out in a fermentation room at 30 °C for 3 h with a thickness of 5 cm, and the relative humidity was over 95% (F). Drying was carried out at 120 °C for 10 min and then at 80 °C for 1 h until it was sufficiently dry (D). Process samples (FL, S1, S2, S3, F) were stored in liquid nitrogen and then freeze-dried.

### 2.2. Reagents and Materials

The fiber of divinylbenzene–carboxen–polydimethylsiloxane (DVB/CAR/PDMS, 50/30 μm) was purchased from Supelco (Bellefonte, PA, USA). The 20 mL headspace vials and screw caps fitted with a silicone/PTFE septum were supplied by Agilent Technologies Inc. (Palo Alto, CA, USA). Ethyl decanoate (AR, internal standard) was purchased from Sigma-Aldrich (Shanghai, China). n-Alkanes (C_3_–C_40_) were used for the determination of retention index (RI) and purchased from Sigma-Aldrich (Shanghai, China).

### 2.3. Sensory Evaluation

Reference materials containing monomers of linalool, hexanal, (*Z*)-hex-3-en-1-yl acetate, β-damascenone, 3-methylbutanal, and 2,5-dimethylpyrazine were used at different concentrations for the descriptive analysis. Prior to evaluation of black tea infusion, panelists were screened and trained to distinguish the flavor of reference materials at various concentrations.

A total of 3.0 g of black tea sample was infused in 150 mL boiled pure water for 5 min in a professional review cup. A 10-point scale based on the method described by Alasalvar et al. [21] was used for scoring. The evaluation, using a linear scale of 0 (none) to 10 (strongest), was completed by five well-trained panelists (two males and three females, aged 25–44 years).

### 2.4. Extraction of Volatiles Using HS-SPME

A total of 0.5 g of ground tea sample was accurately weighed and transferred into a 20 mL headspace vial and infused with 5 mL of boiling water, and then 2 μL of ethyl caprate (7.5 µg/g, 50% ethanol) was added. The vial was kept in a water bath at 60 °C to equilibrate for 5 min, and then the DVB/CAR/PDMS (50/30 μm) SPME fiber was exposed for 40 min to the headspace while the sample was maintained at 60 °C. After adsorption, the extraction head was automatically inserted into the GC × GC–TOFMS injector, and desorption was carried out at 250 °C for 5 min. Each sample was measured in triplicate.

### 2.5. GC × GC–TOFMS Analysis

The volatile analysis was carried out on an Agilent 7890B GC combined with time-of-flight mass spectrometer (TOFMS) detection (LECO Pegasus 4D, Leco Corporation, St. Joseph, MI, USA). Rxi-5Sil (30 m × 250 µm × 0.25 µm; Restek, Bellefonte, PA, USA) and RXi-17Sil MS (1.9 m × 100 µm × 0.10 µm; Restek, Bellefonte, PA, USA) were employed for the first-dimensional and second-dimensional separation, respectively. High purity helium (99.999%) was used as carrier gas with a constant flow of 1.0 mL/min. The temperature of the transfer line was set at 250 °C in splitless mode, and the modulation period was 5.00 s. The initial temperature of the 1st D column was 50 °C for 2 min, and then it was ramped to 265 °C at 8 °C/min holding for 5 min. The temperature of the 2nd D column was initially set at 55 °C for 2 min, and increased to 270 °C at 8 °C/min, and held for 5 min. The mass spectrometer operated at an ion source temperature of 220 °C with a scan mass range of 33–500 *m*/*z*, and the electronic energy was −70 eV. The interface temperature was 270 °C.

### 2.6. Compound Identification

LECO ChromaTOF software was used to process GC × GC–TOFMS data. The peak width of 1st D and 2nd D were set at 25 and 0.1 s, respectively. The minimum signal-to-noise ratio (S/N) was set at 50. Compounds were identified by comparing the similarity of fragments with NIST 11 and Wiley 11 databases, and the minimum similarity match was 750. The RI was calculated by the n-alkane standards (C_3_–C_40_) and compared with that reported in references. The semi-quantitative method with internal standard (ethyl decanoate) was used for relative quantification of volatile compounds. The relative quantification of volatile compounds with internal standard (IS) was calculated using the following equation: the relative concentration of volatile compounds = (volatile compounds peak area/IS peak area) × IS concentration.

### 2.7. rOAV Calculation

The relative odor activity values (rOAVs) of the volatile compounds were calculated by the ratio of relative concentration and its odor threshold in water of each compound.

### 2.8. Statistical Analysis

Significant differences between means were analyzed by one-way ANOVA using SPSS (version 26.0, SPSS Inc., Chicago, IL, USA). The score scatter plot and permutation plot for the OPLS-DA were performed by SIMCA (V14.1, Sartorius Stedim Data Analytics AB, Umea, Sweden). HCA (hierarchical clustering analysis) was drawn using an integrative toolkit—TBtools. The bar charts were generated using Origin 2018 (OriginLab, Northampton, MA, USA).

## 3. Results and Discussion

### 3.1. Changes in Aroma Profile during Shaken Black Tea Processing

**Figure 1:**
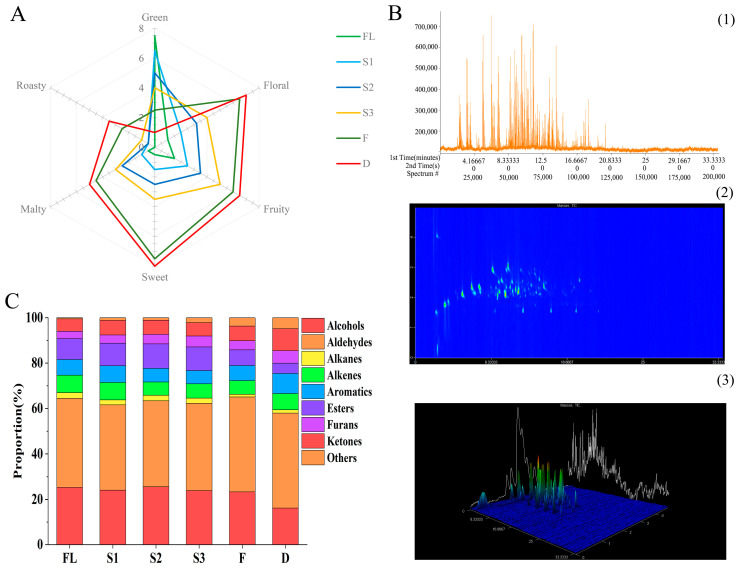
Volatile profiling of shaken black tea leaves from the six processing stages using GC × GC–TOFMS and descriptive analysis. (**A**) Aroma intensity radar map; (**B**) the total ion chromatogram plots of the shaken black tea ((1) 1D contour plots, (2) 2D contour plots, (3) 3D contour plots). (**C**) Aroma-type histogram (fresh leaves (FL), first shaking (S1), second shaking (S2), third shaking (S3), fermentation (F), and drying (D) stages).

Quantitative sensory description analysis was based on the organoleptic properties of black tea infusions using six aroma descriptors: green, floral, fruity, sweet, malty, and roasty (Figure 1A). The sensory evaluation scores of tea samples greatly differed. Statistical analysis showed that six aroma attributes were significantly different when comparing FL with D (*p* < 0.01). The trend in aroma profile was green → fruity and floral fragrance → sweet and floral fragrance. In addition, fruity and floral scents were significantly enhanced during the shaking stage (S1–S3), which reflected the effect of shaking on the formation of the black aroma characteristics. Ultimately, sweet and floral fragrance was the typical aroma characteristic of SBT.

### 3.2. Dynamic Changes in Volatile Metabolites during Shaken Black Tea Processing

Headspace solid-phase microextraction (HS-SPME) combined with GC × GC–TOFMS was used to detect black tea processing samples, and the total number of volatile compounds identified in FL, S1, S2, S3, F, and D were 212, 213, 218, 219, 236, and 241, respectively (Tables S1 and S2). The 1D, 2D, and 3D contour plots of total ion chromatography (TIC) of shaken black tea are shown in Figure 1B. A total of 241 volatile substances was divided into 9 categories, including 49 aldehydes (5 terpene aldehydes), 40 esters, 29 alcohols (10 terpene alcohols), 34 ketones (9 terpene ketones), 30 aromatics, 24 alkenes (14 terpene alkenes), 17 alkanes, 13 furans, and 5 other compounds.

Among them, aldehydes had the highest content of volatile substances, accounting for 37.53–41.76% of total volatile organic compounds (TVOCs). Alcohols were the second most abundant volatiles, accounting for 16.21% to 25.59% of TVOCs, and the proportion decreased significantly during black tea processing, especially in the drying stage (Figure 1C). After drying, the proportion of ketones in TVOCs significantly increased by 76.42% (*p* < 0.01) compared with FL. However, the proportion of alcohol and ester contents in finished tea significantly reduced by 35.98% (*p* < 0.01) and 50.93% (*p* < 0.01), respectively, compared with FL. The proportion of aldehyde, alkene, and aromatic contents were relatively stable during processing. According to the literature, the main aroma components of black tea are alcohols and aldehydes [22], which is consistent with the results of this experiment.

Among tea secondary metabolites, terpenoids are one of the main abundant and diverse classes of natural compounds [23]. The aroma-related volatile terpenoids in teas are mainly terpene alcohols, terpene ketones, terpene alkenes, and terpene aldehydes, which are important components of VOCs in all kinds of finished oolong tea and black tea [24]. A total of 38 terpenoids, terpene alcohols (103.23–137.62 µg/L), and terpene alkenes (24.61–55.47 µg/L) were present in high concentrations. The total amount of terpenoids tended to increase during processing. After drying, terpenoids accounted for 15.63% of TVOCs, an increase of 67.17% over the FL. Terpene alcohols and terpene ketones were identified as the key volatile contributors to the flower and sweet attributes of tea due to an extremely low odor threshold. The thresholds of (E)-β-ionone, β-damascenone, and linalool were 0.007, 0.002, and 6 µg/L, respectively [25], with floral, sweet, and other aromas [26].

### 3.3. Key Odor Contributors to Shaken Black Tea Infusion

To estimate the odor contribution of individual aroma components, their rOAVs were calculated. The rOAVs of volatile compounds identified using GC × GC–TOFMS (Table 1) showed several average rOAVs greater than 1. A total of 27 volatiles had an rOAV ≥ 1 in SBT, and among them, 7 odorants had an rOAV > 20 including (E)-β-ionone, (E,Z)-2,6-nonadienal, 1-octen-3-one, decanal, (Z)-4-heptenal, nonanal, and 2-methyl-butanal. (E)-β-ionone (rOAV 316.07–2073.61) is an important contributor to the aroma of black tea, followed by (E,Z)-2,6-nonadienal (rOAV 74.70–684.89), which is similar to the results of previous studies [25,27]. (E,Z)-2,6-Nonadienal has a cucumber odor and has been identified as one of the important active odor components of Darjeeling black tea [27].

Most of the aroma active components showed an increasing trend mainly during the fermentation and drying stages, and compared with fresh leaves, 19 components increased by more than 50%. 2-Methyl-butanal increased the most, 108 times that of fresh leaves, followed by dimethyl sulfide (26 times) and (E,E)-2,4-decadienal (25 times). Most significantly grown volatiles were found to present a fresh, sweet, and floral odor [25,26]; thus, the increase in grown volatiles may contribute to the formation of the fresh, sweet, and floral aroma quality of SBT.

### 3.4. Analysis of the Volatile Compounds by Multivariate Data Analysis

**Figure 2:**
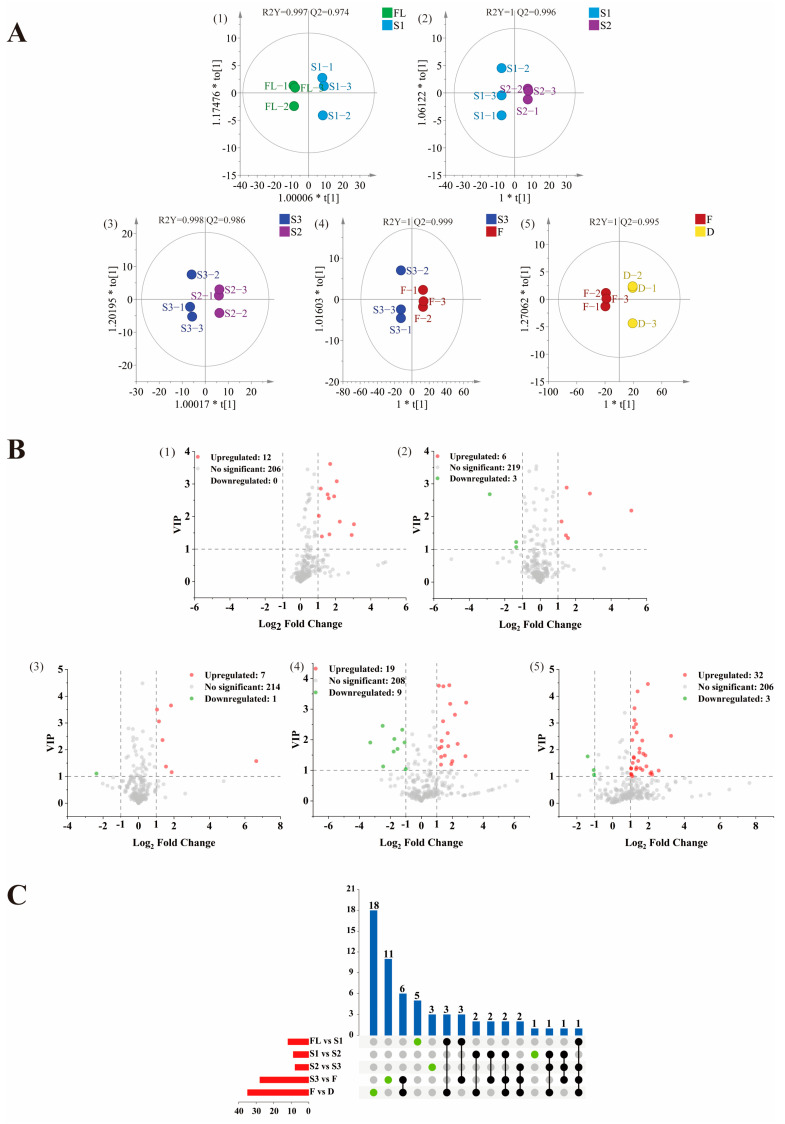
Score scatter plot for the OPLS-DA model (**A**), volcano plot (**B**) and upset plot (**C**) of differential compound among groups FL vs. S1, S1 vs. S2, S2 vs. S3, S3 vs. F, F vs. D. The differential compounds were screened out based on the criteria of variable importance in projection (VIP) > 1, *p* < 0.05, and |log2FC| > 1. In volcano plots, the red dots represent upregulated differential substances, green dots represent downregulated differential substances, and gray dots represent non-significant substances. In upset plot, the red bar chart represents the total number of different substances in groups; the blue bar chart represents the number of different substances shared in groups.

OPLS-DA is a supervised multivariate statistical analysis method, which requires pre-setting classification information and quantifying the contribution of compounds to classification differences through in-depth data mining. OPLS-DA analysis can filter orthogonal variables that are not related to categorical variables and provide more information on the impact of processes on VOCs. Therefore, in order to clarify the influence of each process on the volatile metabolites in each stage of SBT processing, the key differences between each process were explored. The supervised OPLS-DA model was used to investigate the differential metabolites. The OPLS-DA models of FL vs. S1, S1 vs. S2, S2 vs. S3, S3 vs. F, and F vs. D all showed a good explained variance and high predictive capability (Figure 2A). In order to access the performance of these established models, a cross-validation with 200 permutation tests was performed. The intercepts of Q^2^ were negative, suggested that there were no over-fittings existing in these models (Figure S1).

A total of 61 differential volatile compounds was selected based on variable importance in projection (VIP) > 1, *p* < 0 0.05, and |log_2_FC| > 1, including 13 alcohols, 14 aldehydes, 1 alkane, 4 alkenes, 7 aromatics, 4 esters, 4 furans, 12 ketones, and 2 other compounds (Table 2). The distribution of these differential substances in all volatile compounds was visualized by volcano plot, 12 upregulated metabolites of FL vs. S1, 9 differential metabolites (6 upregulated and 3 downregulated) of S1 vs. S2, 8 differential metabolites (7 upregulated and 1 downregulated) of S2 vs. S3, 28 differential metabolites (19 upregulated and 9 downregulated) of S3 vs. F, 35 differential metabolites (32 upregulated and 3 downregulated) of F vs. D were observed, respectively (Figure 2B).

The analysis of the upset plot (Figure 2C) showed that there were numerical overlaps and differences in the differential volatile compounds, indicating that there were common and unique volatile compounds presented between different comparison groups. Several specific compounds can be detected in each comparison group, indicating that these different volatile components were significantly affected by the processing. There were 18, 11, 5, 3, and 1 unique differential metabolites in F vs. D, S3 vs. F, FL vs. S1, S2 vs. S3, and S1 vs. S2, respectively. For example, dimethyl sulfide is an upregulated differential compound in four groups (FL vs. S1, S2 vs. S3, S3 vs. F, F vs. D), indicating that its content was affected by most of the processes during the whole process of SBT processing.

**Figure 3:**
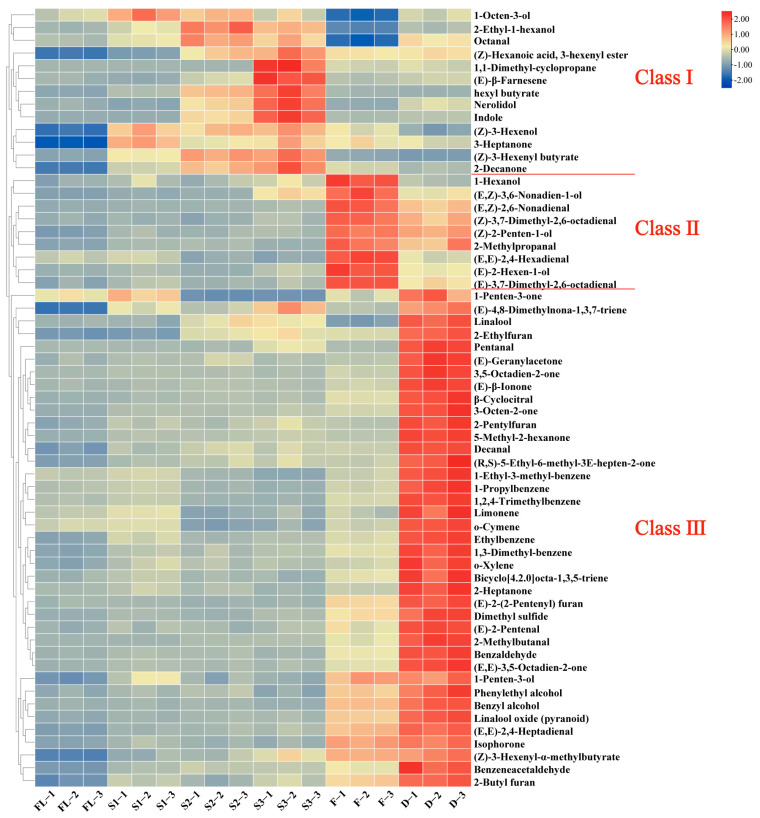
A total of 61 volatile compounds were shown on the heat map, selected by VIP > 1, *p* < 0.05, and |log_2_FC| > 1.

Furthermore, the HCA analysis of the differential metabolites was conducted (Figure 3), in which the color of intensity normalized scale from red to blue indicated the content of compounds from high to low. These differential volatile compounds were assigned to three classes based on the dendrogram: 13 compounds in Class I, 9 compounds in Class II, and 39 compounds in Class III.

The compounds in Class I mainly have high content in the shaking stage, including 1-octen-3-ol, indole, etc. 1-Octen-3-ol is an oxidation degradation product of linoleic acid with cucumber and mushroom aroma [33] and is one of the characteristic aroma components of black tea [34]. Studies have shown that wounding stress during the shaking process activates CsTSB2 and induces the accumulation of indole [12]. Indole is the main aroma component of Oriental Beauty tea and plays a positive role in forming its unique aroma quality [12]. The compounds in Class II showed the highest content in the fermentation process, such as (E,Z)-3,6-nonadien-1-ol, (E,Z)-2,6-nonadienal, 2-methylpropanal, (E,E)-2,4-hexadienal, etc. (E,Z)-3,6-nonadien-1-ol exhibited sweet, green, fruity odor [35]; (E,Z)-2,6-nonadienal showed cucumber odor [27]; 2-methylpropanal had a kind of malty odor [30]; (E,E)-2,4-hexadienal had a sweet, green aroma [35]. The compounds in Class III exhibited the highest content in the drying process, including (E)-β-ionone, decanal, dimethyl sulfide, 2-pentylfuran, 2-ethylfuran, (E,E)-2,4-heptadienal, etc. (E)-β-Ionone had floral odor [31]; decanal and 2-pentylfuran showed fruity odor [4,36]; dimethyl sulfide exhibited cabbage odor [37]; 2-ethylfuran had a kind of caramel-like aroma [38]; (E,E)-2,4-heptadienal had fatty, green odor [35]. These components play a positive role in the formation of aroma quality of SBT.

### 3.5. Effects of Shaking Process on Volatile Aroma Components

A total of 26 different substances was shared in FL vs. S1, S1 vs. S2, and S2 vs. S3, including 5 alcohols, 3 aldehydes, 1 alkane, 2 alkenes, 4 heterocycles, 4 esters, 2 furans, 3 ketones, and 2 other classes (Table 2). Except for 1-propylbenzene, 1-ethyl-3-methyl-benzene, and 1-penten-3-one, most of the components were upregulated and provided a green, sweet, floral, and cocoa odor [39].

More specifically, benzeneacetaldehyde, (Z)-3-hexenol, 1,3-dimethyl-benzene, dimethyl sulfide, (Z)-3-hexenyl butyrate, ethylbenzene, 1-penten-3-ol, (E)-4,8-dimethylnona-1,3,7-triene, 3-heptanone, (Z)-3-hexenyl-α-methylbutyrate, 2-decanone, and 2-butylfuran were shown significantly upregulated in FL vs. S1 group. Benzeneacetaldehyde was formed by the Strecker degradation reaction of phenylalanine, which exhibited a rose-like odor [26] and is a very important flavor compound in various teas, especially in oolong tea and black tea [40,41]. Dimethyl sulfide showed a cabbage odor [37] and is considered a beneficial compound to the aroma of green tea and oolong tea [24]. Ethylbenzene represented an aromatic odor and was one of the most definite odorants contributed to the green tea with chestnut-like [42]. (Z)-3-Hexenol (green, grass, fruity odor) [40], (E)-4,8-dimethylnona-1,3,7-triene, 3-heptanone (green pepper) [43], and (Z)-3-hexenyl-α-methylbutyrate (fruity odor like unripe apple and pineapple) [44] were the specific differential compounds only in FL vs. S1 and mostly showed green and fruity odor, indicating that the first shaking process was the key process for the accumulation of these substances. Studies have shown that benzeneacetaldehyde, dimethyl sulfide significantly increased during the shaking process of oolong tea [24,45]. Moreover, it has been reported that the accumulation of (Z)-3-hexenol during the shaking process was a stable phenomenon, due to the de novo synthesis pathway, rather than enzymatic hydrolysis [46,47].

In the S1 vs. S2 group, six compounds were upregulated, including 2-ethylfuran, 2-methylbutanal, indole, nerolidol, (Z)-hexanoic acid, 3-hexenyl ester, and hexyl butyrate, and three compounds (1-penten-3-one, 1-ethyl-3-methyl-benzene and 1-propylbenzene) were downregulated. The upregulated compounds mainly exhibited sweet, floral, and fruity aroma [26,27,39]. Studies have shown that the accumulation of (Z)-hexanoic acid, 3-hexenyl ester occurred in the shaking process [48] and exhibited a fruity and green odor [25]. The results of this experiment were consistent with the literature [48], and further proved that the second shaking was the key process, which was conducive to the accumulation of (Z)-hexanoic acid, 3-hexenyl ester.

In the S2 vs. S3 group, there were seven upregulated differential metabolites (pentanal, dimethyl sulfide, indole, (E)-β-farnesene, etc.) and one downregulated differential metabolite (1-ethyl-3-methyl-benzene). Among them, 1,1-dimethyl-cyclopropane, (E)-β-farnesene, and (E,Z)-3,6-nonadien-1-ol (sweet, green, waxy, melon, fruity) [35] were the special metabolites. (E)-β-Farnesene exhibited floral odor [42], the content of which was 2.92-fold higher in S3 than that in S2.

The leaves were the breathing tissue even after picked from the tea tree to the end of the shaking stage, which could respond to the stress (mechanical damage, dehydration) during tea processing. Indole was a characteristic aroma component of oolong tea, and previous studies have shown that the accumulation of indole occurred mainly in the shaking stage of oolong tea [12,24]. This experimental study was consistent with the results of the existing literature [12,24], and it was proven that the accumulation of indole was mainly in the second and third shaking stages (S2, S3).

### 3.6. Effects of Fermentation Process on Volatile Aroma Components

In S3 vs. F group, 19 of these volatiles were significantly upregulated, including dimethyl sulfide, (E,E)-2,4-heptadienal, benzaldehyde, benzyl alcohol, etc. The other nine volatiles were significantly downregulated, including 1-octen-3-ol, nerolidol, indole, etc.

Among the above 28 differential compounds, 13 substances belonged to fatty acid degradation products. The fresh tea leaves were crushed by rolling, and the unsaturated fatty acids underwent enzymatic hydrolysis to form C_6_–C_9_ alcohols, aldehydes, ketones, and esters [33]. During fermentation, most alcohols ((E)-2-hexen-1-ol, (Z)-2-penten-1-ol, 1-penten-3-ol) and aldehydes ((E,Z)-2,6-nonadienal, (E,E)-2,4-heptadienal) were upregulated, while esters ((Z)-3-hexenyl butyrate, hexyl butyrate) were downregulated. Most of these upregulated fatty acid degradation products had the aroma characteristics of fresh, fruity, and sweet aroma [4,33], which contributed to the presentation of the aroma quality of black tea.

Four volatile components were associated with amino acid degradation, including benzaldehyde, benzyl alcohol, 2-methylbutyraldehyde, and 2-methylpropanal, and these aroma components were all upregulated during the fermentation process. These substances presented floral, fruity, and delicate aromas [4,7], and the increase in their content might contribute to the formation of the floral and fruity aromas of black tea. Benzaldehyde and benzyl alcohol were products of the shikimate pathway, which were produced by phenylalanine under the action of phenylalanine decarboxylase [49]. They had sweet, fruity, and citrus-like aromas and were reported to be the main aldehyde in black tea [50].

During fermentation, metabolites of the amino acid metabolic pathway increased significantly, and free amino acids were confirmed to be degraded to volatile compounds in black tea [26,34]. Both phenylethyl alcohol and benzeneacetaldehyde were biosynthesized via the phenylalanine metabolism pathway. Phenylethyl alcohol with honey, spice, rose, and lilac smell and benzeneacetaldehyde with honey and sweet flavor were very important flavor compounds in various teas [26]. Isoleucine and valine underwent Strecker degradation to form 2-methylbutanal and 2-methylpropanal [33], respectively, which were the main reasons for the increase of these two volatile aroma components during the fermentation process of black tea. It could be seen that the upregulated volatile compounds during the fermentation of black tea were mainly fatty acids and amino acid degradation products, which presented sweet, floral, and fruity flavor. The aroma substances formed in these fermentation processes laid a certain foundation for the formation of the flavor quality of black tea.

### 3.7. Effects of Drying Process on Volatile Aroma Components

In F vs. D groups, 33 of these volatiles were significantly upregulated, including β-cyclocitral, (E)-β-ionone, and (E)-geranylacetone, etc. β-Cyclocitral had lemon and fruity aromas [31], while (E)-geranylacetone had fresh, rose, and floral aromas [35]. (E)-β-Ionone exhibited floral, woody, and berry aromas and had a low odor threshold (0.007 μg/L) [31], which played an important role in the presentation of black tea flavors and was driven by enzymatic oxidation during (E)-β-carotene fermentation or thermal degradation during drying [24,51]. In this study, the enrichment of (E)-β-ionone occurred mainly during the drying process and reached a maximum value (14.52 µg/L) 3.34 times that of the fermented leaves. This indicated that the pyrolysis of carotenoids to form (E)-β-ionone was more severe compared with the enzymatic degradation.

In addition, 1-octen-3-ol (cucumber, mushroom), pentanal (almond, malt), (E)-2-pentenal (strawberry, fruit), octanal (fatty, lemon, fragrance), decanal (orange peel odor), and 1-penten-3-one (onion odor) were all derived from unsaturated fatty acid metabolic pathways, because unsaturated fatty acids were oxidized and degraded during the drying process [33,34]. Moreover, 2-pentylfuran had fruity, green, earthy, bean, and vegetable flavors and was a substance that had an important contribution to fruity aroma [36]. The formation of 2-pentylfuran is associated with the Maillard reaction and Strecker degradation of sugars and amino acids [33]. These aroma substances had certain promoting effects on the appearance of sweet and fruity aroma of finished tea.

## 4. Conclusions

GC × GC–TOFMS was used to detect the dynamic aroma changes of SBT during manufacture, and a total of 241 volatile components was finally identified. (E)-β-ionone, (E,Z)-2,6-nonadienal, 1-octen-3-one, decanal, (Z)-4-heptenal, nonanal, and 2-methyl-butanal made significant contributions to the aroma profile of SBT due to their high rOAVs. The OPLS-DA models of FL vs. S1, S1 vs. S2, S2 vs. S3, S3 vs. F, and F vs. D were established, respectively, and these models had good reliability. A total of 61 discriminatory volatile compounds (VIP > 1, *p* < 0.05, and |log_2_FC > 1|) were screened out, of which 26 substances were shared in common among FL vs. S1, S1 vs. S2, and S2 vs. S3. The key components upregulated in the shaking stage were (Z)-3-hexenol, (Z)-hexanoic acid, 3-hexenyl ester, (E)-β-farnesene, and indole, which promoted the formation of the floral and fruity flavor of black tea. The volatile compounds in the fermentation stage were mainly fatty acids and amino acid degradation products and presented sweet, floral, and fruity flavor, which laid the material foundation for the formation of the flavor of black tea. The content of amino acid sources or the volatile components formed by the Maillard reaction in which they participated was significantly increased during the drying stage, and these components had a direct effect on the aroma quality of black tea. This study provides the dynamic changes of VOCs during SBT processing and the influence of processes on their changes, which is of great significance to improve the flavor quality of black tea and lay a solid foundation for the promotion of SBT production technology.

## Figures and Tables

**Table 1:** The relative odor activity values (rOAVs) of the most important aroma-active compounds during the manufacture of shaken black tea.

No.	Compounds	Threshold (μg/kg) ^a^	The Relative Odor Activity Values (rOAVs)					
			FL	S1	S2	S3	F	D
235	(E)-β-Ionone	0.007 [25]	316.07	351.81	424.80	387.76	583.74	2073.61
64	(E,Z)-2,6-Nonadienal	0.01 [25]	74.70	100.04	105.23	134.88	684.89	409.65
208	1-Octen-3-one	0.005 [25]	196.58	280.05	241.43	233.60	191.88	301.46
67	Decanal	0.1 [25]	33.07	47.22	55.08	52.86	53.02	108.41
46	(Z)-4-Heptenal	0.06 [27]	31.00	36.75	33.54	38.39	22.61	20.76
61	Nonanal	1 [25]	19.04	22.77	22.94	23.05	15.30	27.23
34	2-Methylbutanal	1 [25]	0.80	0.73	5.05	6.16	22.40	87.52
27	Geraniol	3.2 [27]	19.97	18.57	20.57	19.10	16.91	22.57
54	Octanal	0.7 [25]	15.72	18.59	24.18	21.48	9.11	19.77
215	(E,E)-3,5-Octadien-2-one	0.5 [28]	4.62	8.01	6.91	6.80	16.73	52.60
39	Hexanal	4.5 [25]	13.42	13.54	11.63	13.58	14.02	22.40
73	(E,E)-2,4-Decadienal	0.07 [25]	1.39	2.92	3.24	7.07	25.40	35.84
65	(E)-2-Nonenal	0.08 [25]	4.67	5.47	6.17	7.35	24.70	20.12
9	1-Octen-3-ol	1 [25]	10.73	15.60	13.90	11.26	5.31	10.84
68	(E,E)-2,4-Nonadienal	0.16 [27]	1.47	3.37	4.55	5.80	14.67	17.18
237	Dimethyl sulfide	3 [25]	0.90	2.63	1.47	3.02	10.55	24.55
45	Heptanal	3 [25]	5.42	6.61	6.48	5.97	4.18	5.97
57	Benzeneacetaldehyde	4 [25]	1.06	3.41	4.25	4.21	6.07	14.11
197	2-Pentylfuran	6 [29]	2.47	3.35	3.17	3.75	3.26	8.03
193	2-Ethylfuran	2.3 [30]	1.01	1.19	3.33	3.83	3.19	7.26
59	(E)-2-Octenal	3 [25]	1.60	2.16	2.20	2.39	4.53	6.40
56	(E,E)-2,4-Heptadienal	10 [25]	1.01	1.61	1.74	1.89	4.14	5.99
20	cis-Linalool oxide (furanoid)	6 [31]	2.06	2.94	2.38	2.40	2.38	2.24
44	2-Hexenal	17 [31]	2.71	2.86	2.43	2.36	2.04	1.65
109	Limonene	10 [25]	1.67	1.95	1.27	1.44	1.65	3.52
233	α-Ionone	0.4 [32]	1.08	0.98	1.23	1.13	1.35	5.11
32	2-Methyl-propanal	0.9 [29]	0.32	0.86	0.88	0.61	4.38	3.27

^a^ The thresholds of compounds referred to the literature.

**Table 2:** A total of 61 differential volatile compounds during the manufacturing of shaken black tea.

No.	Compounds	FL vs. S1			S1 vs. S2			S2 vs. S3			S3 vs. F			F vs. D		
		Log_2_FC	VIP	Type	Log_2_FC	VIP	Type	Log_2_FC	VIP	Type	Log_2_FC	VIP	Type	Log_2_FC	VIP	Type
**Alcohols (13)**																
2	1-Penten-3-ol	1.05	2.02	Up							1.15	1.72	Up			
4	(Z)-2-Penten-1-ol										1.70	2.22	Up			
5	(Z)-3-Hexenol	2.06	3.08	Up												
6	1-Hexanol													−1.06	1.24	Down
7	(E)-2-Hexen-1-ol							1.35	2.36	Up	2.17	2.82	Up	−1.40	1.75	Down
9	1-Octen-3-ol										−1.09	1.91	Down	1.03	1.30	Up
10	2-Ethyl-1-hexanol										−1.75	2.03	Down			
11	Benzyl alcohol										2.90	3.21	Up			
14	Phenylethyl alcohol										1.40	2.60	Up			
16	(E,Z)-3,6-Nonadien-1-ol							1.87	1.16	Up						
22	Linalool													1.53	1.31	Up
23	Linalool oxide (Pyranoid)										1.51	1.48	Up			
28	Nerolidol				1.20	1.85	Up				−1.79	1.61	Down			
**Aldehydes (14)**																
32	2-Methylpropanal										2.85	1.46	Up			
34	2-Methylbutanal				2.80	2.71	Up				1.86	3.17	Up	1.97	4.46	Up
36	Pentanal							1.83	3.66	Up	−1.54	1.70	Down	3.26	2.52	Up
37	(E)-2-Pentenal													1.02	1.09	Up
48	(E,E)-2,4-Hexadienal										1.73	1.79	Up	−1.04	1.06	Down
52	Benzaldehyde										1.42	3.75	Up	1.39	4.18	Up
54	Octanal										−1.24	2.33	Down	1.12	1.51	Up
56	(E,E)-2,4-Heptadienal										1.13	3.77	Up			
57	Benzeneacetaldehyde	1.69	3.62	Up										1.22	3.11	Up
64	(E,Z)-2,6-Nonadienal										2.34	1.86	Up			
67	Decanal													1.03	1.30	Up
76	β-Cyclocitral													1.31	1.27	Up
77	(Z)-3,7-Dimethyl-2,6-octadienal; (Z)-Citral										1.27	1.19	Up			
78	(E)-3,7-Dimethyl-2,6-octadienal; Geranial										1.31	1.96	Up			
**Alkanes (1)**																
79	1,1-Dimethyl-cyclopropane							6.64	1.58	Up						
**Alkenes (4)**																
98	Bicyclo [4.2.0]octa-1,3,5-triene													1.18	2.84	Up
102	(E)-4,8-Dimethylnona-1,3,7-triene	2.23	1.84	Up												
109	Limonene													1.09	2.36	Up
116	(E)-β-Farnesene							1.55	1.37	Up						
**Aromatics (7)**																
121	Ethylbenzene	1.61	2.56	Up										1.50	2.04	Up
122	1,3-Dimethyl-benzene	1.15	2.86	Up										1.34	2.65	Up
124	o-Xylene													1.33	1.34	Up
125	1-Propylbenzene				−1.36	1.07	Down							2.10	1.11	Up
126	1-Ethyl-3-methyl-benzene				−1.36	1.23	Down	−2.38	1.11	Down				2.16	1.16	Up
128	1,2,4-Trimethylbenzene													1.66	1.24	Up
129	o-Cymene													1.18	1.73	Up
**Esters (4)**																
163	(Z)-3-hexenyl butyrate	1.92	2.62	Up							−2.50	2.46	Down			
164	Hexyl butyrate				1.57	1.35	Up				−2.47	1.13	Down			
170	(Z)-3-Hexenyl-α-methylbutyrate	1.65	1.46	Up	_	_	_									
182	(Z)-Hexanoic acid, 3-hexenyl ester				1.45	1.43	Up									
**Furans (4)**																
193	2-Ethylfuran				1.49	2.89	Up							1.19	1.69	Up
196	2-Butylfuran	1.22	1.39	Up												
197	2-Pentylfuran,													1.30	2.96	Up
198	(E)-2-(2-Pentenyl)furan										2.00	1.30	Up	1.05	1.08	Up
**Ketones (12)**																
203	1-Penten-3-one				−2.85	2.68	Down				1.94	1.21	Up	1.09	1.01	Up
205	3-Heptanone	3.04	1.76	Up												
206	2-Heptanone													1.88	1.38	Up
212	3-Octen-2-one													1.71	1.85	Up
214	Isophorone										1.28	1.44	Up			
215	(E,E)-3,5-Octadien-2-one										1.30	1.77	Up	1.65	2.34	Up
219	3,5-Octadien-2-one													2.57	1.22	Up
221	5-Methyl-2-hexanone													2.21	1.07	Up
222	(R,S)-5-Ethyl-6-methyl-3E-hepten-2-one													1.49	1.90	Up
223	2-Decanone	2.91	1.44	Up							−1.01	1.04	Down			
234	(E)-Geranylacetone													1.42	1.58	Up
235	(E)-β-Ionone													1.83	1.79	Up
**Other (2)**																
237	Dimethyl sulfide	1.54	2.68	Up				1.04	3.50	Up	1.81	3.78	Up	1.22	3.55	Up
241	Indole				5.14	2.18	Up	1.15	3.06	Up	−3.30	1.91	Down			

## Data Availability

No new data were created or analyzed in this study. Data sharing is not applicable to this article.

## Supplementary Materials

The following supporting information can be downloaded at: https://www.mdpi.com/article/10.3390/foods11091228/s1, Table S1: Relative contents of the volatile compounds in shaken black tea samples during the manufacture based on GC × GC-TOFMS; Table S2: Characterization of the volatile compounds in shaken black tea samples during the manufacture based on GC × GC-TOFMS; Figure S1: (A1) permutation plot for the OPLS-DA model (FL vs. S1); (A2) permutation plot for the OPLS-DA model (S1 vs. S2); (A3) permutation plot for the OPLS-DA model (S3 vs. S2); (A4) permutation plot for the OPLS-DA model (S3 vs. F); (A5) permutation plot for the OPLS-DA model (F vs. D).
