# Supplementary material for: Dynamic Changes in Volatile Compounds of Shaken Black Tea during Its Manufacture by GC × GC–TOFMS and Multivariate Data Analysis
Source: Foods. 2022 Apr 25;11(9):1228. doi: 10.3390/foods11091228 (PMC9102106; doi:10.3390/foods11091228)
Supplement: Supplementary file 1 [file foods-11-01228-s001.zip › foods-1647152-supplementary.pdf]

Supporting Information for

Dynamic changes in volatile compounds of shaken black tea during its manufacture by GC × GC-TOFMS and multivariate data analysis

Jinjin Xue [1,2,#], Panpan Liu [3,#], Junfeng Yin [1], Weiwei Wang [1], Jianyong Zhang [1], Wei Wang [1], Ting Le [1], Dejiang Ni [2,*], Heyuan Jiang[1,*],

[1] Key Laboratory of Tea Biology and Resources Utilization, Ministry of Agriculture, Tea Research Institute, Chinese Academy of Agricultural Sciences, Hangzhou, 310008, China

[2] Huazhong Agricultural University, Wuhan, 430070, China

[3] Fruit and Tea Research Institute, Hubei Academy of Agricultural Sciences, Wuhan, Hubei, 430064, China

*Corresponding Author

Email addresses: nidj@mail.hzau.edu.cn; jianghy@tricaas.com

[#] These authors contributed equally to this work.

Table S1. Relative contents of the volatile compounds in shaken black tea samples during the manufacture based on GC × GC-TOFMS.

No.	Compounds	Precursors	RT[a] (min)	RT[b] (s)	Relative contents (μg/L)					
					FL	S1	S2	S3	F	D
**Alcohols (19)**										
1	2-Methyl-2-propanol		2.50	1.44	-	0.27±0.01	0.14±0.01	0.13±0.01	0.08±0.00	0.31±0.09
2	1-Penten-3-ol	Lipids	3.50	1.75	2.83±0.21 ^{c}	5.85±0.91 ^{b}	4.08±0.67 ^{c}	3.89±0.22 ^{c}	8.64±0.81 ^{a}	9.84±0.93 ^{a}
3	1-Pentanol	Lipids	4.58	1.93	0.32±0.05 ^{d}	0.50±0.03 ^{cd}	0.62±0.08 ^{bc}	0.75±0.05 ^{b}	0.69±0.05 ^{bc}	3.19±0.28 ^{a}
4	(Z)-2-Penten-1-ol	Lipids	4.67	2.01	1.69±0.17 ^{d}	3.22±0.40 ^{c}	3.03±0.31 ^{c}	3.44±0.31 ^{c}	11.18±0.41 ^{a}	9.24±0.59 ^{b}
5	(Z)-3-Hexenol	Lipids; Glycosides	6.25	2.19	2.17±0.22 ^{d}	9.07±0.96 ^{a}	8.93±0.93 ^{a}	9.66±0.84 ^{a}	7.10±0.60 ^{b}	4.39±0.52 ^{c}
6	1-Hexanol	Lipids	6.42	2.09	4.03±0.34 ^{c}	4.92±0.68 ^{bc}	4.47±0.49 ^{c}	5.43±0.56 ^{b}	9.63±0.34 ^{a}	4.62±0.25 ^{bc}
7	(E)-2-Hexen-1-ol	Lipids	6.42	2.15	1.71±0.17 ^{e}	2.57±0.24 ^{d}	1.40±0.07 ^{e}	3.58±0.55 ^{c}	16.12±0.94 ^{a}	6.11±0.26 ^{b}
8	1-Heptanol	Lipids	8.58	2.11	1.90±0.17 ^{ab}	2.09±0.18 ^{a}	1.69±0.10 ^{b}	1.32±0.10 ^{c}	0.46±0.02 ^{e}	0.79±0.07 ^{d}
9	1-Octen-3-ol	Lipids	8.75	2.1	10.73±0.71 ^{c}	15.60±1.29 ^{a}	13.90±0.54 ^{b}	11.26±1.40 ^{c}	5.31±0.23 ^{d}	10.84±0.58 ^{c}
10	2-Ethyl-1-hexanol		9.75	2.1	4.92±0.11 ^{d}	6.83±0.60 ^{c}	11.43±0.90 ^{a}	9.19±0.28 ^{b}	2.74±0.16 ^{e}	5.36±0.26 ^{d}
11	Benzyl alcohol	Glycosides	10.00	2.9	2.75±0.33 ^{c}	3.47±0.56 ^{c}	3.77±0.38 ^{c}	2.52±0.28 ^{c}	18.75±0.26 ^{b}	31.25±2.08 ^{a}
12	(E)-2-Octen-1-ol		10.58	2.21	0.26±0.02 ^{b}	0.27±0.02 ^{ab}	0.29±0.02 ^{a}	0.26±0.02 ^{b}	0.17±0.01 ^{c}	0.28±0.01 ^{ab}
13	1-Methylcycloheptanol		11.50	2.51	0.31±0.03 ^{c}	0.45±0.02 ^{b}	0.46±0.06 ^{b}	0.41±0.05 ^{bc}	0.46±0.00 ^{b}	1.79±0.12 ^{a}
14	Phenylethyl alcohol	Glycosides	11.58	2.92	6.83±0.95 ^{d}	8.59±0.65 ^{cd}	9.70±0.69 ^{c}	6.55±1.05 ^{d}	17.32±0.71 ^{b}	27.33±3.04 ^{a}
15	(Z)-3-Nonen-1-ol		12.25	2.23	0.19±0.02 ^{d}	0.38±0.02 ^{c}	0.43±0.04 ^{bc}	0.47±0.03 ^{b}	0.63±0.03 ^{a}	0.40±0.05 ^{c}
16	(E,Z)-3,6-Nonadien-1-ol		12.42	2.31	0.08±0.00 ^{c}	0.17±0.02 ^{c}	0.19±0.01 ^{c}	0.70±0.07 ^{b}	1.36±0.12 ^{a}	0.58±0.07 ^{a}
17	1-Nonanol	Lipids	12.58	2.14	5.23±0.68 ^{a}	5.00±0.09 ^{ab}	4.56±0.19 ^{b}	3.73±0.35 ^{c}	2.48±0.03 ^{d}	2.65±0.16 ^{d}
18	1-Decanol		14.50	2.11	0.39±0.03 ^{b}	0.44±0.04 ^{a}	0.45±0.04 ^{a}	0.28±0.01 ^{c}	0.20±0.02 ^{d}	0.19±0.01 ^{d}
19	Z-2-Dodecenol		16.83	2.13	0.05±0.00 ^{d}	0.06±0.01 ^{d}	0.11±0.01 ^{b}	0.09±0.00 ^{c}	0.06±0.01 ^{d}	0.18±0.01 ^{a}
**Terpene alcohols (10)**										
20	(Z)-Linalool oxide (Furanoid)	Glycosides	10.67	2.23	12.35±0.96 ^{b}	17.65±1.10 ^{a}	14.29±1.32 ^{b}	14.42±1.99 ^{b}	14.31±0.47 ^{b}	13.44±0.67 ^{b}
21	(E)-Linalool oxide (Furanoid)	Glycosides	11.00	2.28	17.28±0.89 ^{d}	22.35±2.67 ^{bc}	19.74±1.40 ^{cd}	20.72±2.04 ^{c}	24.12±0.64 ^{ab}	26.73±1.21 ^{a}
22	Linalool	Glycosides	11.25	2.35	3.60±0.10 ^{c}	3.50±0.27 ^{cd}	5.18±0.59 ^{b}	5.28±0.23 ^{b}	2.95±0.17 ^{d}	8.55±0.31 ^{a}
23	Linalool oxide (Pyranoid)	Glycosides	12.67	2.43	1.07±0.13 ^{d}	1.58±0.12 ^{c}	1.85±0.15 ^{c}	1.87±0.23 ^{c}	5.34±0.13 ^{b}	9.65±0.57 ^{a}

No.	Compounds	Precursors	RT[a] (min)	RT[b] (s)	Relative contents (μg/L)					
					FL	S1	S2	S3	F	D
24	Terpinen-4-ol		12.92	2.34	-	-	-	0.06±0.01	0.15±0.00	0.12±0.01
25	α-Terpineol	Glycosides	13.17	2.39	0.29±0.04 ^{c}	0.37±0.03 ^{b}	0.35±0.03 ^{b}	0.36±0.05 ^{b}	0.45±0.00 ^{a}	0.38±0.01 ^{b}
26	(Z)-3,7-Dimethyl-2,6-octadien-1-ol; Nerol		13.67	2.31	2.87±0.30 ^{d}	3.10±0.10 ^{cd}	3.18±0.12 ^{cd}	3.38±0.26 ^{bc}	3.82±0.10 ^{a}	3.64±0.20 ^{ab}
27	Geraniol	Glycosides	14.08	2.43	63.89±5.81 ^{abc}	59.44±5.11 ^{bc}	65.83±1.15 ^{ab}	61.12±8.74 ^{bc}	54.12±4.43 ^{c}	72.22±3.30 ^{a}
28	Nerolidol	Carotenoids	19.25	2.21	1.81±0.11 ^{d}	1.57±0.06 ^{d}	3.60±0.24 ^{b}	5.80±0.44 ^{a}	1.68±0.03 ^{d}	2.77±0.17 ^{c}
29	Cedrol		20.17	2.61	0.08±0.01 ^{d}	0.08±0.01 ^{d}	0.19±0.01 ^{a}	0.12±0.01 ^{c}	0.05±0.00 ^{e}	0.14±0.00 ^{b}
**Aldehydes (44)**										
30	Acetaldehyde	Maillard reaction	2.08	3.93	0.23±0.05 ^{bc}	0.12±0.01 ^{cd}	0.10±0.01 ^{d}	0.20±0.04 ^{bcd}	0.30±0.05 ^{b}	0.65±0.12 ^{a}
31	2-Propenal		2.25	4.08	-	-	0.11±0.02	0.30±0.02	0.39±0.05	0.37±0.08
32	2-Methylpropanal	Glycosides	2.50	4.1	0.28±0.04 ^{c}	0.77±0.02 ^{c}	0.79±0.06 ^{c}	0.55±0.01 ^{c}	3.95±0.37 ^{a}	2.94±0.81 ^{b}
33	Butanal		2.67	0.79	0.47±0.04 ^{d}	0.75±0.07 ^{c}	0.25±0.05 ^{e}	0.30±0.01 ^{e}	0.86±0.03 ^{b}	1.21±0.01 ^{a}
34	2-Methylbutanal	Maillard reaction	3.17	1.73	0.80±0.03 ^{c}	0.73±0.07 ^{c}	5.05±0.36 ^{c}	6.16±0.29 ^{c}	22.40±3.51 ^{b}	87.52±5.99 ^{a}
35	(E)-2-Butenal		3.25	1.88	0.20±0.02 ^{b}	0.17±0.03 ^{b}	0.51±0.65 ^{b}	0.15±0.02 ^{b}	0.52±0.08 ^{b}	1.96±0.09 ^{a}
36	Pentanal	Lipids	3.67	1.85	2.07±0.14 ^{c}	2.00±0.16 ^{c}	1.97±0.33 ^{c}	7.00±0.61 ^{b}	2.41±0.17 ^{c}	23.09±1.03 ^{a}
37	(E)-2-Pentenal		4.42	2.17	2.18±0.15 ^{c}	2.60±0.33 ^{c}	2.40±0.23 ^{c}	2.66±0.24 ^{c}	3.82±0.51 ^{b}	7.77±0.08 ^{a}
38	3-Hexenal	Lipids	5.08	2.19	3.94±0.32 ^{a}	3.96±0.24 ^{a}	2.91±0.14 ^{b}	2.43±0.33 ^{c}	1.67±0.24 ^{d}	1.30±0.06 ^{d}
39	Hexanal	Lipids	5.17	2.11	60.38±2.71 ^{b}	60.92±2.72 ^{b}	52.34±3.51 ^{c}	61.10±4.51 ^{b}	63.09±1.77 ^{b}	100.79±4.49 ^{a}
40	2-Ethyl-3-methyl-butanal		5.83	2.04	0.17±0.01 ^{c}	0.33±0.03 ^{b}	0.10±0.01 ^{c}	0.07±0.01 ^{c}	0.10±0.01 ^{c}	0.90±0.18 ^{a}
41	Furfural	Maillard reaction	5.83	2.78	-	-	-	-	-	1.88±0.08 ^{a}
42	(E)-2-Hexenal	Lipids	6.00	2.29	4.96±0.41 ^{a}	4.36±0.32 ^{b}	2.88±0.11 ^{d}	2.81±0.26 ^{d}	3.64±0.28 ^{c}	1.86±0.04 ^{e}
43	3-Furaldehyde		6.17	2.66	-	-	-	-	-	0.40±0.01
44	2-Hexenal	Lipids	6.25	2.34	46.04±1.79 ^{a}	48.60±1.32 ^{a}	41.28±1.41 ^{b}	40.16±1.99 ^{b}	34.60±2.91 ^{c}	28.09±1.16 ^{d}
45	Heptanal	Lipids	7.08	2.2	16.26±0.42 ^{c}	19.84±0.55 ^{a}	19.43±0.63 ^{a}	17.91±1.01 ^{b}	12.53±0.46 ^{d}	17.90±0.89 ^{b}
46	(Z)-4-Heptenal		7.08	2.29	1.86±0.06 ^{c}	2.20±0.18 ^{ab}	2.01±0.15 ^{bc}	2.30±0.09 ^{a}	1.36±0.10 ^{d}	1.25±0.07 ^{d}
47	Methional	Maillard reaction	7.25	2.94	-	-	-	-	0.40±0.02	1.02±0.07
48	(E,E)-2,4-Hexadienal	Lipids	7.42	2.56	3.13±0.22 ^{b}	3.30±0.19 ^{b}	2.28±0.20 ^{c}	2.17±0.17 ^{c}	7.22±0.24 ^{a}	3.50±0.30 ^{b}

No.	Compounds	Precursors	RT[a] (min)	RT[b] (s)	Relative contents (μg/L)					
					FL	S1	S2	S3	F	D
49	2,2-Dimethyl-3,4-pentadienal		8.00	2.05	0.58±0.08 ^{d}	0.99±0.08 ^{c}	0.70±0.06 ^{d}	0.56±0.09 ^{d}	2.13±0.05 ^{b}	3.69±0.20 ^{a}
50	2-Ethylhexanal		8.25	2.09	0.41±0.04 ^{c}	0.52±0.05 ^{b}	0.42±0.04 ^{c}	0.27±0.02 ^{d}	0.29±0.02 ^{d}	0.61±0.02 ^{a}
51	(Z)-2-Heptenal		8.33	2.33	1.71±0.11 ^{d}	2.25±0.19 ^{c}	2.16±0.03 ^{c}	2.41±0.08 ^{c}	3.69±0.12 ^{b}	6.35±0.33 ^{a}
52	Benzaldehyde	Glycosides	8.50	2.89	12.09±0.91 ^{e}	16.73±0.91 ^{c}	16.43±1.03 ^{cd}	13.13±1.49 ^{de}	35.23±0.58 ^{b}	92.32±4.07 ^{a}
53	(E,Z)-2,4-Heptadienal	Lipids	9.17	2.51	10.81±0.48 ^{d}	18.61±0.54 ^{c}	17.64±0.27 ^{c}	19.86±1.93 ^{c}	33.52±1.48 ^{b}	43.55±2.16 ^{a}
54	Octanal		9.33	1.99	11.00±0.26 ^{d}	13.01±0.30 ^{c}	16.93±0.82 ^{a}	15.04±1.25 ^{b}	6.38±0.49 ^{e}	13.84±0.49 ^{bc}
55	2-Ethyl-2-hexenal		9.33	2.32	0.10±0.00 ^{b}	0.09±0.01 ^{b}	0.08±0.01 ^{c}	0.07±0.01 ^{c}	0.05±0.00 ^{d}	0.18±0.02 ^{a}
56	(E,E)-2,4-Heptadienal	Lipids	9.50	2.55	10.05±0.51 ^{d}	16.07±0.84 ^{c}	17.40±1.54 ^{c}	18.91±2.07 ^{c}	41.40±2.00 ^{b}	59.89±2.29 ^{a}
57	Benzeneacetaldehyde	Maillard reaction	10.17	2.98	4.23±0.42 ^{d}	13.63±1.03 ^{c}	16.99±1.49 ^{c}	16.83±0.48 ^{c}	24.28±1.75 ^{b}	56.45±5.35 ^{a}
58	Aldehydes, 2-(1-pentenyl)-, (E)-		10.33	2.24	0.04±0.00 ^{c}	0.04±0.00 ^{c}	0.03±0.00 ^{c}	0.04±0.00 ^{c}	0.13±0.01 ^{b}	0.36±0.04 ^{a}
59	(E)-2-Octenal		10.42	2.33	4.79±0.36 ^{d}	6.47±0.48 ^{c}	6.61±0.34 ^{c}	7.16±0.47 ^{c}	13.58±0.46 ^{b}	19.20±0.49 ^{a}
60	2-Methylbenzaldehyde		10.67	1.7	0.30±0.04 ^{d}	0.46±0.03 ^{bc}	0.48±0.04 ^{b}	0.42±0.01 ^{c}	0.28±0.03 ^{d}	0.80±0.04 ^{a}
61	Nonanal	Lipids	11.33	2.22	19.04±1.02 ^{c}	22.77±0.34 ^{b}	22.94±1.52 ^{b}	23.05±1.78 ^{b}	15.30±0.47 ^{d}	27.23±2.37 ^{a}
62	(E,E)-2,4-Octadienal		11.50	2.49	-	-	-	0.26±0.02	0.69±0.00	1.31±0.15
63	2-Methylenehexanal		11.92	1.73	0.39±0.04 ^{c}	0.39±0.05 ^{c}	0.57±0.06 ^{b}	0.57±0.05 ^{b}	0.17±0.01 ^{d}	1.40±0.07 ^{a}
64	(E,Z)-2,6-Nonadienal	Lipids	12.25	2.43	0.75±0.07 ^{d}	1.00±0.06 ^{cd}	1.05±0.07 ^{cd}	1.35±0.09 ^{c}	6.85±0.45 ^{a}	4.10±0.33 ^{b}
65	(E)-2-Nonenal	Lipids	12.42	2.29	0.37±0.04 ^{e}	0.44±0.03 ^{de}	0.49±0.03 ^{d}	0.59±0.04 ^{c}	1.98±0.06 ^{a}	1.61±0.05 ^{b}
66	2,4-dimethyl-Benzaldehyde		12.83	2.81	-	-	-	-	0.11±0.01	0.97±0.05
67	Decanal		13.25	2.17	3.31±0.25 ^{d}	4.72±0.27 ^{c}	5.51±0.26 ^{b}	5.29±0.42 ^{b}	5.30±0.05 ^{b}	10.84±0.15 ^{a}
68	(E,E)-2,4-Nonadienal		13.50	2.45	0.24±0.03 ^{f}	0.54±0.02 ^{e}	0.73±0.06 ^{d}	0.93±0.09 ^{c}	2.35±0.08 ^{b}	2.75±0.13 ^{a}
69	2,6,6-Trimethyl-1-cyclohexene-1-acetaldehyde		14.25	2.51	0.54±0.02 ^{e}	0.68±0.03 ^{d}	0.80±0.06 ^{c}	0.71±0.07 ^{d}	0.99±0.01 ^{b}	2.09±0.05 ^{a}
70	α-Ethylidene-benzeneacetaldehyde		14.58	2.15	0.17±0.01 ^{d}	0.42±0.02 ^{c}	0.53±0.03 ^{b}	0.28±0.04 ^{d}	0.22±0.00 ^{d}	1.85±0.14 ^{a}
71	(E,Z)-2,4-Decadienal		14.92	2.38	0.10±0.00 ^{d}	0.21±0.00 ^{c}	0.23±0.01 ^{c}	0.32±0.04 ^{c}	1.57±0.05 ^{b}	1.71±0.13 ^{a}
72	Undecanal		15.08	2.16	0.19±0.01 ^{d}	0.27±0.01 ^{c}	0.34±0.03 ^{b}	0.30±0.03 ^{c}	0.28±0.02 ^{c}	0.49±0.01 ^{a}
73	(E,E)-2,4-Decadienal	Lipids	15.42	2.41	0.10±0.00 ^{d}	0.20±0.01 ^{d}	0.23±0.02 ^{d}	0.49±0.07 ^{c}	1.78±0.06 ^{b}	2.51±0.17 ^{a}
**Terpene aldehydes (5)**										

No.	Compounds	Precursors	RT[a] (min)	RT[b] (s)	Relative contents (μg/L)					
					FL	S1	S2	S3	F	D
74	α-Cyclocitral	Carotenoids	11.67	2.41	0.11±0.01 d	0.15±0.01 c	0.13±0.01 cd	0.11±0.01 d	0.36±0.01 b	0.73±0.04 a
75	2,6,6-Trimethyl-1,3-cyclohexadiene-1-carboxaldehyde; Safranal	Carotenoids	13.25	2.67	0.10±0.01 c	0.12±0.01 bc	0.14±0.01 bc	0.14±0.01 bc	0.21±0.00 b	2.40±0.14 a
76	β-Cyclocitral	Carotenoids	13.58	2.62	1.86±0.06 d	2.19±0.13 cd	2.47±0.10 c	2.40±0.14 c	3.59±0.04 b	8.87±0.47 a
77	(Z)-3,7-Dimethyl-2,6-octadienal; (Z)-Citral		13.92	2.47	1.33±0.09 c	1.39±0.06 c	1.30±0.11 c	1.58±0.13 c	3.81±0.17 a	2.88±0.28 b
78	(E)-3,7-Dimethyl-2,6-octadienal; geranial		14.42	2.49	3.33±0.39 d	4.30±0.32 c	3.76±0.16 cd	4.09±0.37 c	10.16±0.02 a	6.19±0.37 b
**Alkanes (17)**										
79	1,1-Dimethyl-cyclopropane		2.33	3.92	-	-	-	0.94±0.19	0.16±0.03	0.17±0.05
80	n-Hexane		2.75	0.24	0.10±0.02 c	0.26±0.03 a	0.05±0.01 d	0.06±0.01 d	0.12±0.02 bc	0.14±0.02 b
81	Octane		5.17	0.65	1.22±0.18 b	1.55±0.25 ab	1.56±0.18 ab	1.68±0.38 a	0.43±0.04 c	0.70±0.07 c
82	Nonane		7.08	1.68	0.15±0.01 bc	0.17±0.01 b	0.13±0.02 bcd	0.10±0.01 cd	0.09±0.01 d	0.40±0.07 a
83	2,2,4,6,6-Pentamethyl-heptane		9.00	1.68	1.12±0.04 cd	1.28±0.05 c	2.11±0.11 a	1.75±0.31 b	0.74±0.06 e	0.99±0.03 d
84	Decane		9.17	1.71	0.82±0.07 c	0.92±0.03 b	0.57±0.07 d	0.62±0.06 d	0.56±0.02 d	1.13±0.03 a
85	Undecane		11.25	1.73	2.48±0.22 c	3.33±0.34 b	2.99±0.29 b	2.52±0.29 c	1.84±0.14 d	4.00±0.01 a
86	5-Methylundecane		11.75	1.76	0.56±0.03 d	0.59±0.02 d	1.09±0.05 b	1.21±0.10 a	0.61±0.04 d	0.91±0.06 c
87	3-Methylundecane		12.58	1.74	0.53±0.03 b	0.53±0.04 b	0.49±0.05 b	0.48±0.07 b	0.39±0.04 c	0.76±0.03 a
88	Dodecane		13.17	1.75	2.49±0.37 b	2.64±0.12 b	2.64±0.12 b	2.60±0.35 b	1.80±0.07 c	3.49±0.25 a
89	Tridecane	Lipids	14.92	1.78	0.90±0.07 c	0.99±0.01 bc	1.04±0.07 b	1.06±0.12 b	0.75±0.09 d	1.57±0.01 a
90	2,6,10-Trimethyl-dodecane		15.50	1.75	0.43±0.04 b	0.36±0.02 c	0.31±0.01 de	0.26±0.02 e	0.33±0.02 cd	0.75±0.04 a
91	3-Methyl-tridecane		16.17	1.77	0.70±0.09 b	0.62±0.04 bc	0.54±0.06 c	0.52±0.09 c	0.59±0.05 bc	1.56±0.08 a
92	Tetradecane		16.67	1.78	1.05±0.10 b	1.00±0.06 bc	0.93±0.06 bc	0.90±0.12 bc	0.83±0.05 c	2.20±0.16 a
93	Pentadecane	Lipids	18.25	1.79	1.06±0.07 c	1.05±0.03 c	1.32±0.12 b	0.98±0.12 c	0.80±0.07 d	1.63±0.11 a
94	3-Methyl-dodecane		19.33	1.79	0.50±0.02 c	0.47±0.04 c	0.68±0.03 b	0.47±0.07 c	0.35±0.06 d	1.04±0.04 a
95	Hexadecane	Lipids	19.75	1.81	0.45±0.07 d	0.40±0.03 d	0.80±0.05 b	0.67±0.05 c	0.39±0.03 d	1.09±0.09 a
**Alkenes (10)**										

No.	Compounds	Precursors	RT[a] (min)	RT[b] (s)	Relative contents (μg/L)					
					FL	S1	S2	S3	F	D
96	(Z)-1,3-Pentadiene		2.42	3.97	0.45±0.08 ^{d}	1.16±0.18 ^{bc}	0.85±0.18 ^{c}	1.59±0.20 ^{a}	1.51±0.40 ^{ab}	1.00±0.10 ^{c}
97	(E,Z)-1,3,5-Octatriene		6.67	1.97	0.22±0.02 ^{c}	0.38±0.02 ^{b}	0.27±0.03 ^{c}	0.13±0.02 ^{d}	0.24±0.02 ^{c}	0.92±0.10 ^{a}
98	Bicyclo[4.2.0]octa-1,3,5-triene		7.00	2.35	11.93±1.42 ^{d}	16.61±1.06 ^{c}	12.87±0.78 ^{cd}	11.83±1.50 ^{d}	21.08±0.84 ^{b}	47.89±4.88 ^{a}
99	(Z)-1-Phenylpropene		8.08	2.3	-	-	-	-	0.06±0.01	0.37±0.04
100	α-Methylstyrene		8.92	2.36	0.04±0.01 ^{cd}	0.05±0.01 ^{c}	0.04±0.00 ^{de}	0.03±0.00 ^{e}	0.12±0.01 ^{b}	0.36±0.01 ^{a}
101	1,4-Hexadiene, 3,3,5-trimethyl-		10.75	1.88	0.14±0.01 ^{de}	0.19±0.01 ^{c}	0.12±0.02 ^{e}	0.17±0.04 ^{cd}	0.25±0.01 ^{b}	0.45±0.03 ^{a}
102	(E)-4,8-Dimethylnona-1,3,7-triene		11.50	2.03	0.66±0.07 ^{e}	3.08±0.27 ^{c}	2.52±0.22 ^{cd}	4.35±0.57 ^{b}	2.42±0.11 ^{d}	5.04±0.40 ^{a}
103	(E)-3-Undecene		13.00	1.8	0.36±0.02 ^{b}	0.40±0.00 ^{ab}	0.41±0.05 ^{ab}	0.35±0.05 ^{b}	0.26±0.04 ^{c}	0.46±0.02 ^{a}
104	2,7,7-Trimethyl-bicyclo[2.2.1]hept-2-ene		13.83	2.34	-	-	-	-	0.23±0.02	0.43±0.03
105	(Z)-3-Tetradecene		16.50	1.84	0.91±0.03 ^{b}	0.78±0.05 ^{c}	0.61±0.07 ^{d}	0.52±0.03 ^{d}	0.63±0.08 ^{d}	1.37±0.12 ^{a}
**Terpene alkenes (14)**										
106	β-Myrcene		9.00	1.97	8.09±0.53 ^{b}	8.47±0.26 ^{b}	6.64±0.56 ^{cd}	6.96±0.79 ^{c}	5.90±0.10 ^{d}	10.15±0.18 ^{a}
107	α-Phellandrene		9.42	2.01	0.41±0.01 ^{b}	0.45±0.02 ^{a}	0.27±0.02 ^{c}	0.13±0.01 ^{d}	0.24±0.01 ^{c}	0.38±0.03 ^{b}
108	γ-Terpinene		9.58	2.03	0.34±0.01 ^{b}	0.38±0.01 ^{a}	0.23±0.02 ^{d}	0.14±0.01 ^{e}	0.27±0.02 ^{c}	0.40±0.01 ^{a}
109	Limonene		9.83	2.1	16.74±0.43 ^{bc}	19.51±0.17 ^{b}	12.69±0.73 ^{d}	14.36±1.50 ^{cd}	16.51±1.54 ^{bc}	35.19±3.32 ^{a}
110	(E)-β-Ocimene		9.92	2.19	0.11±0.00 ^{d}	0.20±0.01 ^{b}	0.16±0.00 ^{c}	0.09±0.00 ^{d}	0.22±0.02 ^{b}	0.52±0.05 ^{a}
111	β-Ocimene		10.17	2.03	3.30±0.32 ^{abc}	3.84±0.18 ^{a}	3.11±0.40 ^{bc}	3.68±0.56 ^{ab}	2.77±0.06 ^{c}	3.51±0.09 ^{ab}
112	β-Phellandrene		10.42	2.12	0.79±0.06 ^{b}	0.78±0.01 ^{b}	0.37±0.04 ^{d}	0.39±0.04 ^{d}	0.53±0.01 ^{c}	2.83±0.08 ^{a}
113	Terpinolene		11.00	2.16	0.32±0.01 ^{b}	0.32±0.02 ^{b}	0.20±0.03 ^{d}	0.22±0.04 ^{d}	0.27±0.01 ^{c}	0.56±0.01 ^{a}
114	β-Elemene		16.58	2.14	0.06±0.01 ^{d}	0.08±0.01 ^{c}	0.07±0.00 ^{c}	0.09±0.01 ^{c}	0.11±0.00 ^{b}	0.28±0.00 ^{a}
115	Cedrene		17.08	2.26	0.29±0.02 ^{c}	0.28±0.04 ^{c}	0.36±0.02 ^{b}	0.40±0.04 ^{b}	0.27±0.01 ^{c}	0.84±0.04 ^{a}
116	(E)-β-Farnesene		17.50	2.08	0.19±0.02 ^{d}	0.15±0.01 ^{d}	0.36±0.03 ^{b}	1.06±0.07 ^{a}	0.26±0.02 ^{c}	0.34±0.02 ^{b}
117	α-Farnesene	Carotenoids	18.33	2.12	0.05±0.00 ^{d}	0.05±0.01 ^{d}	0.08±0.01 ^{c}	0.16±0.01 ^{b}	0.09±0.01 ^{c}	0.20±0.01 ^{a}
118	Alloaromadendrene		18.42	2.35	0.02±0.00 ^{c}	0.02±0.00 ^{c}	0.04±0.00 ^{b}	0.04±0.00 ^{b}	0.03±0.00 ^{c}	0.15±0.01 ^{a}
119	δ-Cadinene		18.67	2.31	0.03±0.00 ^{c}	0.03±0.00 ^{bc}	0.03±0.00 ^{bc}	0.04±0.00 ^{b}	0.03±0.00 ^{bc}	0.14±0.01 ^{a}
**Aromatics (30)**										

No.	Compounds	Precursors	RT[a] (min)	RT[b] (s)	Relative contents (μg/L)					
					FL	S1	S2	S3	F	D
120	Toluene		4.67	0.97	1.89±0.24 [b]	2.24±0.53 [b]	2.15±0.46 [b]	1.87±0.46 [b]	2.49±0.43 [b]	5.76±0.18 [a]
121	Ethylbenzene		6.33	2.17	2.28±0.38 [e]	6.96±0.60 [b]	4.06±0.17 [d]	4.93±0.08 [c]	7.45±0.33 [b]	21.04±0.79 [a]
122	1,3-Dimethyl-benzene		6.50	2.14	4.75±0.50 [d]	10.58±0.74 [c]	9.13±1.76 [c]	10.04±0.39 [c]	14.98±0.37 [b]	37.99±2.30 [a]
123	p-Xylene		6.58	1.56	0.07±0.01 [e]	0.10±0.01 [de]	0.13±0.04 [d]	0.28±0.02 [b]	0.18±0.01 [c]	0.55±0.02 [a]
124	o-Xylene		7.00	2.25	2.02±0.20 [c]	3.60±0.58 [b]	3.12±0.31 [b]	3.12±0.18 [b]	3.87±0.22 [b]	9.72±0.67 [a]
125	1-Propylbenzene		8.25	2.19	0.74±0.09 [c]	1.12±0.08 [b]	0.44±0.06 [cd]	0.29±0.05 [d]	1.22±0.09 [b]	5.26±0.42 [a]
126	1-Ethyl-3-methyl-benzene		8.42	2.2	1.07±0.05 [c]	1.46±0.05 [b]	0.57±0.05 [d]	0.11±0.01 [e]	1.26±0.14 [bc]	5.65±0.35 [a]
127	(1-Methylethyl)-benzene		8.75	2.29	0.39±0.04 [c]	0.49±0.02 [b]	0.27±0.01 [d]	0.07±0.00 [e]	0.41±0.02 [c]	1.30±0.05 [a]
128	1,2,4-Trimethylbenzene		9.17	2.26	1.48±0.08 [d]	1.93±0.06 [c]	1.03±0.05 [e]	0.98±0.12 [e]	2.34±0.07 [b]	7.39±0.29 [a]
129	o-Cymene		9.75	2.2	7.94±0.45 [bc]	8.51±0.08 [b]	4.52±0.27 [e]	5.29±0.70 [d]	7.72±0.29 [c]	17.44±0.48 [a]
130	1,2,3-Trimethylbenzene		9.75	2.37	0.68±0.03 [c]	0.91±0.03 [b]	0.56±0.05 [d]	0.47±0.07 [d]	0.92±0.02 [b]	2.75±0.12 [a]
131	Indane		10.00	2.54	0.23±0.03 [c]	0.31±0.02 [b]	0.18±0.01 [d]	0.17±0.01 [d]	0.25±0.01 [c]	0.74±0.03 [a]
132	1-Methyl-3-propyl-benzene		10.42	2.22	0.22±0.01 [c]	0.23±0.01 [c]	0.15±0.01 [d]	0.15±0.03 [d]	0.32±0.02 [b]	1.11±0.05 [a]
133	2-Ethyl-1,4-dimethyl-benzene		11.00	2.32	0.55±0.07 [b]	0.55±0.03 [b]	0.40±0.04 [d]	0.42±0.05 [cd]	0.49±0.01 [bc]	1.57±0.05 [a]
134	1-Methyl-4-(1-methylethenyl)-benzene		11.08	2.38	0.39±0.03 [c]	0.39±0.03 [c]	0.35±0.04 [c]	0.55±0.03 [b]	1.05±0.10 [a]	1.07±0.13 [a]
135	1,2,3,4-Tetramethyl-benzene		11.67	2.37	1.59±0.08 [c]	1.61±0.06 [c]	1.06±0.06 [d]	1.15±0.13 [d]	1.88±0.17 [b]	4.22±0.15 [a]
136	2,3-Dihydro-4-methyl-1H-indene		12.08	2.52	0.24±0.02 [c]	0.26±0.01 [c]	0.14±0.02 [d]	0.16±0.03 [d]	0.30±0.00 [b]	0.80±0.03 [a]
137	2-Ethenyl-1,4-dimethyl-benzene		12.25	2.58	0.44±0.02 [c]	0.44±0.04 [c]	0.26±0.04 [d]	0.30±0.04 [d]	0.52±0.00 [b]	1.09±0.03 [a]
138	Pentylbenzene		12.42	2.22	0.05±0.00 [c]	0.04±0.00 [d]	0.03±0.00 [d]	0.03±0.00 [e]	0.08±0.01 [b]	0.24±0.00 [a]
139	1,2,3,4-Tetrahydronaphthalene		12.58	2.65	0.43±0.04 [b]	0.40±0.03 [b]	0.24±0.01 [c]	0.28±0.04 [c]	0.43±0.01 [b]	0.76±0.04 [a]
140	(1,1-Dimethylpropyl)benzene		12.75	2.28	0.15±0.01 [bc]	0.13±0.03 [c]	0.07±0.01 [d]	0.09±0.01 [d]	0.17±0.02 [b]	0.46±0.03 [a]
141	1-Methyl-4-(1-methyl-2-propenyl)-benzene		13.00	2.44	0.25±0.03 [b]	0.25±0.01 [b]	0.15±0.02 [c]	0.18±0.03 [c]	0.28±0.02 [b]	0.59±0.03 [a]
142	1,1-Dimethyl-2,3-dihydro-1H-indene		13.17	2.49	0.22±0.01 [b]	0.20±0.01 [b]	0.12±0.01 [d]	0.16±0.03 [c]	0.21±0.02 [b]	0.35±0.03 [a]
143	1,2,3,4-Tetrahydro-2-methylnaphthalene		13.58	2.56	0.23±0.02 [c]	0.23±0.01 [c]	0.18±0.03 [d]	0.20±0.02 [cd]	0.33±0.01 [b]	0.51±0.04 [a]
144	1-Methyl-1,2,3,4-		13.75	2.6	0.14±0.02 [b]	0.13±0.01 [b]	0.10±0.01 [c]	0.10±0.01 [c]	0.13±0.00 [b]	0.19±0.01 [a]

No.	Compounds	Precursors	RT[a] (min)	RT[b] (s)	Relative contents (μg/L)					
					FL	S1	S2	S3	F	D
	tetrahydronaphthalene									
145	1,3,3-Trimethyltricyclo[2.2.1.0(2,6)]heptane		14.08	2.45	11.26±0.79 [b]	10.73±0.58 [bc]	12.67±1.14 [a]	10.59±0.98 [bc]	9.35±0.32 [c]	10.41±0.15 [bc]
146	2,3-Dihydro-1,3-dimethyl-1H-indene		14.17	2.54	0.19±0.01 [bc]	0.20±0.00 [b]	0.17±0.01 [cd]	0.16±0.02 [d]	0.21±0.00 [b]	0.39±0.02 [a]
147	1,2,3,4-tetrahydro-5-methyl-naphthalene		14.50	2.62	0.37±0.03 [b]	0.31±0.01 [c]	0.26±0.00 [d]	0.22±0.03 [e]	0.35±0.02 [b]	0.51±0.03 [a]
148	1-Methyl-naphthalene		15.08	2.92	0.38±0.06 [b]	0.41±0.03 [b]	0.38±0.05 [b]	0.25±0.04 [c]	0.46±0.04 [b]	0.95±0.06 [a]
149	1,2,3,4-Tetrahydro-1,4-dimethyl-naphthalene		15.42	2.53	0.15±0.01 [d]	0.19±0.01 [bc]	0.16±0.01 [cd]	0.15±0.02 [d]	0.21±0.01 [b]	0.25±0.01 [a]
**Esters (40)**										
150	Hexanoic acid, methyl ester		7.58	2.1	0.08±0.01 [d]	0.23±0.03 [a]	0.19±0.01 [b]	0.07±0.01 [d]	0.13±0.01 [c]	0.22±0.02 [a]
151	Ethyl hexanoate		9.08	2.1	0.55±0.03 [d]	0.92±0.08 [b]	0.75±0.10 [c]	0.80±0.09 [bc]	0.84±0.03 [bc]	1.81±0.08 [a]
152	Allyl methallyl ether		9.17	2.24	0.47±0.05 [b]	0.55±0.09 [b]	0.55±0.03 [b]	0.20±0.00 [d]	0.30±0.00 [c]	0.68±0.05 [a]
153	Acetic acid, hexyl ester	Lipids	9.42	2.1	0.31±0.03 [c]	0.63±0.06 [a]	0.54±0.06 [b]	0.49±0.05 [b]	0.12±0.00 [e]	0.21±0.01 [d]
154	(Z)-3-Hexen-1-ol, acetate	Lipids	9.50	2.13	0.38±0.07 [a]	0.42±0.06 [a]	0.10±0.01 [b]	0.11±0.01 [b]	0.07±0.01 [b]	0.09±0.01 [b]
155	2-Hexenoic acid, ethyl ester		10.08	2.49	0.11±0.01 [d]	0.19±0.02 [b]	0.15±0.00 [c]	0.12±0.01 [d]	0.11±0.00 [d]	0.21±0.01 [a]
156	Formic acid, phenylmethyl ester		10.83	2.82	-	-	-	-	0.07±0.00	0.18±0.01
157	Heptanoic acid, ethyl ester		11.08	2.11	0.06±0.01 [d]	0.11±0.01 [c]	0.05±0.00 [d]	0.07±0.01 [d]	0.15±0.01 [b]	0.54±0.03 [a]
158	Methyl benzoate		11.17	2.79	1.19±0.10 [cd]	1.29±0.05 [c]	1.07±0.09 [d]	0.78±0.13 [e]	1.56±0.07 [b]	3.13±0.15 [a]
159	Octanoic acid, methyl ester		11.67	2.11	0.10±0.01 [e]	0.24±0.02 [cd]	0.21±0.03 [d]	0.25±0.02 [c]	0.36±0.02 [b]	0.44±0.02 [a]
160	(E)-Butanoic acid, 3-hexenyl ester	Lipids	12.00	1.89	0.07±0.00 [d]	0.15±0.00 [b]	0.16±0.01 [b]	0.21±0.02 [a]	0.11±0.01 [c]	0.16±0.01 [b]
161	Acetic acid, 2-ethylhexyl ester		12.08	2.07	3.33±0.17 [bc]	3.63±0.20 [bc]	3.79±0.44 [b]	3.47±0.41 [bc]	3.14±0.12 [c]	4.67±0.22 [a]
162	Acetic acid, phenylmethyl ester	Amino acids	12.50	2.8	0.49±0.02 [c]	0.52±0.06 [c]	0.66±0.07 [c]	0.45±0.06 [c]	1.44±0.06 [b]	4.01±0.26 [a]
163	(Z)-3-hexenyl-butyrate	Lipids	12.83	2.16	1.77±0.13 [c]	6.67±0.15 [b]	10.76±0.68 [a]	11.68±1.48 [a]	2.06±0.21 [c]	1.26±0.05 [c]
164	Hexyl butyrate		12.92	2.09	0.22±0.01 [d]	0.55±0.02 [c]	1.62±0.09 [b]	2.48±0.32 [a]	0.45±0.05 [cd]	0.38±0.01 [cd]
165	Octanoic acid, ethyl ester		13.00	2.09	0.21±0.02 [d]	0.30±0.01 [c]	0.37±0.05 [c]	0.53±0.08 [b]	0.59±0.03 [b]	0.95±0.03 [a]
166	Methyl salicylate	Glycosides	13.08	2.83	29.03±2.27 [bc]	38.40±2.52 [a]	32.42±2.77 [b]	30.73±3.20 [b]	25.43±0.41 [c]	14.68±0.58 [d]

No.	Compounds	Precursors	RT[a] (min)	RT[b] (s)	Relative contents (μg/L)					
					FL	S1	S2	S3	F	D
167	Nonanoic acid, methyl ester		13.50	1.81	0.05±0.00 ^{c}	0.06±0.00 ^{c}	0.06±0.00 ^{c}	0.07±0.00 ^{c}	0.14±0.01 ^{b}	0.53±0.04 ^{a}
168	2-Methyl-pentanoic acid, methyl ester		13.50	2.28	0.64±0.02 ^{e}	0.85±0.08 ^{d}	1.07±0.06 ^{c}	1.10±0.06 ^{bc}	1.27±0.08 ^{b}	3.20±0.19 ^{a}
169	2-Ethylhexyl acrylate		13.58	2.12	10.94±0.41 ^{b}	11.06±0.40 ^{b}	13.23±0.21 ^{a}	11.35±0.61 ^{b}	8.64±0.20 ^{c}	12.70±0.58 ^{a}
170	(Z)-3-Hexenyl-α-methylbutyrate	Lipids, amino acids	13.67	2.12	0.72±0.06 ^{e}	2.27±0.38 ^{d}	2.66±0.40 ^{d}	4.13±0.63 ^{c}	5.82±0.06 ^{b}	6.83±0.62 ^{a}
171	Hexyl 2-methylbutyrate		13.75	2.06	0.03±0.00 ^{f}	0.08±0.01 ^{e}	0.11±0.01 ^{d}	0.21±0.02 ^{c}	0.59±0.01 ^{b}	1.05±0.01 ^{a}
172	(Z)-3-Hexenyl isovalerate		13.83	2.09	0.14±0.00 ^{d}	0.18±0.01 ^{c}	0.17±0.00 ^{c}	0.21±0.01 ^{b}	0.10±0.01 ^{e}	2.11±0.01 ^{a}
173	(E)-2-Hexenyl isovalerate		13.92	2.03	0.66±0.04 ^{bc}	0.65±0.03 ^{c}	0.77±0.06 ^{b}	0.71±0.12 ^{bc}	0.60±0.02 ^{c}	1.13±0.05 ^{a}
174	Acetic acid, 2-phenylethyl ester	Amino acids	14.17	2.78	0.07±0.00 ^{d}	0.09±0.00 ^{c}	0.09±0.01 ^{c}	0.07±0.01 ^{d}	0.12±0.00 ^{b}	0.24±0.01 ^{a}
175	Benzoic acid, 2-hydroxy-, ethyl ester		14.50	2.68	0.07±0.00 ^{e}	0.09±0.01 ^{d}	0.07±0.01 ^{de}	0.14±0.00 ^{c}	0.55±0.01 ^{a}	0.30±0.02 ^{b}
176	Hexanoic acid, pentyl ester		14.67	1.81	0.23±0.02 ^{c}	0.55±0.03 ^{b}	0.48±0.04 ^{b}	0.51±0.05 ^{b}	0.49±0.02 ^{b}	0.68±0.06 ^{a}
177	Nonanoic acid, ethyl ester		14.83	2.08	0.10±0.00 ^{c}	0.14±0.01 ^{bc}	0.18±0.02 ^{b}	0.18±0.04 ^{b}	0.17±0.01 ^{b}	0.97±0.08 ^{a}
178	n-Butyric acid 2-ethylhexyl ester		15.25	2.05	0.92±0.08 ^{c}	0.95±0.04 ^{c}	1.40±0.06 ^{a}	1.02±0.07 ^{bc}	1.03±0.03 ^{bc}	1.10±0.05 ^{b}
179	Decanoic acid, methyl ester		15.33	2.1	0.14±0.01 ^{c}	0.18±0.02 ^{b}	0.15±0.01 ^{c}	0.11±0.01 ^{d}	0.40±0.01 ^{a}	0.11±0.01 ^{d}
180	Methyl geranate		15.33	2.32	0.69±0.08 ^{de}	0.90±0.07 ^{c}	0.65±0.04 ^{e}	0.78±0.06 ^{d}	1.70±0.06 ^{b}	1.90±0.08 ^{a}
181	Dihydrocarvyl acetate		15.83	1.92	0.03±0.01 ^{b}	0.03±0.01 ^{bc}	0.03±0.00 ^{bc}	0.03±0.00 ^{bc}	0.02±0.00 ^{c}	0.15±0.01 ^{a}
182	(Z)-Hexanoic acid, 3-hexenyl ester	Lipids	16.25	2.16	0.24±0.03 ^{d}	0.71±0.04 ^{c}	1.95±0.24 ^{b}	2.52±0.35 ^{a}	1.75±0.05 ^{b}	1.80±0.06 ^{b}
183	Hexanoic acid, hexyl ester	Lipids	16.33	2.09	-	0.03±0.01	0.12±0.00	0.16±0.01	0.16±0.00	0.24±0.01
184	(Z,Z)-3-Hexenoic acid, 3-hexenyl ester	Lipids	16.33	2.24	-	0.06±0.01	0.11±0.00	0.13±0.01	0.10±0.01	0.11±0.00
185	Linalyl isobutyrate		16.33	2.3	0.09±0.01 ^{e}	0.18±0.02 ^{b}	0.16±0.02 ^{bc}	0.14±0.00 ^{c}	0.11±0.01 ^{d}	0.22±0.01 ^{a}
186	Hexanoic acid, 2-hexenyl ester, (E)-		16.42	2.17	0.03±0.01 ^{e}	0.09±0.00 ^{d}	0.25±0.02 ^{c}	0.34±0.02 ^{b}	0.62±0.02 ^{a}	0.64±0.05 ^{a}
187	(Z)-3-Hexen-1-ol, benzoate		19.42	2.67	-	0.04±0.00	0.45±0.07	0.65±0.00	0.49±0.05	0.90±0.06
188	Benzoic acid, hexyl ester		19.58	2.53	-	-	-	-	0.10±0.01	0.25±0.02
189	Hippuric acid, methyl ester		19.67	2.66	-	-	-	-	0.22±0.02	0.45±0.02
**Furans (13)**										
190	Furan		2.33	3.98	-	-	-	-	0.06±0.01	0.20±0.01

No.	Compounds	Precursors	RT[a] (min)	RT[b] (s)	Relative contents (μg/L)					
					FL	S1	S2	S3	F	D
191	2-Methylfuran		2.75	4.14	-	0.24±0.03	0.32±0.02	0.47±0.06	0.53±0.07	2.40±0.14
192	Tetrahydrofuran		3.08	0.41	-	0.20±0.02	0.20±0.02	0.40±0.06	0.33±0.01	0.76±0.07
193	2-Ethylfuran		3.67	1.79	2.31±0.30 [d]	2.73±0.26 [d]	7.66±0.37 [c]	8.80±1.03 [b]	7.34±0.20 [c]	16.70±0.64 [a]
194	2-Ethyl-5-methyl-furan		5.08	1.99	-	-	-	-	-	0.28±0.03
195	2-Methoxy-furan		5.08	2.19	-	-	-	-	-	0.86±0.04
196	2-Butyl furan		6.92	2.01	1.05±0.18 [e]	2.44±0.13 [c]	1.87±0.21 [d]	2.55±0.29 [c]	3.76±0.19 [b]	5.76±0.20 [a]
197	2-Pentylfuran	Maillard reaction	9.00	2.08	14.83±0.98 [d]	20.10±1.20 [bc]	19.03±1.36 [c]	22.51±2.07 [b]	19.57±0.56 [c]	48.19±1.99 [a]
198	(E)-2-(2-Pentenyl)furan		9.17	2.19	1.00±0.18 [c]	1.01±0.01 [c]	0.82±0.08 [c]	0.88±0.10 [c]	3.55±0.09 [b]	7.33±0.36 [a]
199	2-(2-Propenyl)furan		9.83	2.76	-	-	-	-	0.31±0.02	0.50±0.01
200	2,5-Furandicarboxaldehyde		9.83	2.77	-	-	-	0.03±0.00	0.14±0.00	0.55±0.04
201	2-Hexyl-furan		11.08	2.07	0.12±0.01 [d]	0.19±0.01 [c]	0.17±0.01 [c]	0.19±0.01 [c]	0.39±0.01 [b]	1.01±0.05 [a]
202	3-Phenylfuran		13.67	2.86	-	-	-	-	0.19±0.01	3.08±0.35
**Ketones (25)**										
203	1-Penten-3-one	Lipids	3.50	1.91	3.83±0.38 [c]	4.98±0.54 [b]	0.69±0.10 [d]	0.84±0.01 [d]	3.21±0.57 [c]	6.85±1.27 [a]
204	2,3-Hexanedione		4.83	2.03	0.25±0.04	0.30±0.05	-	-	0.12±0.02	0.59±0.07
205	3-Heptanone		6.75	2.15	0.31±0.02 [d]	2.54±0.16 [a]	1.91±0.10 [c]	2.31±0.32 [ab]	2.03±0.17 [bc]	1.76±0.16 [c]
206	2-Heptanone		6.83	2.16	1.56±0.08 [c]	2.29±0.35 [b]	1.79±0.20 [bc]	1.35±0.12 [c]	2.32±0.07 [b]	8.55±0.54 [a]
207	6-Methyl-2-heptanone		8.17	2.15	1.07±0.09 [cd]	1.48±0.14 [b]	1.18±0.13 [c]	0.85±0.05 [e]	0.96±0.03 [de]	2.36±0.05 [a]
208	1-Octen-3-one	Lipids	8.67	2.26	0.98±0.05 [c]	1.40±0.03 [a]	1.21±0.03 [b]	1.17±0.08 [b]	0.96±0.06 [c]	1.51±0.09 [a]
209	2,5-Octanedione		8.83	2.18	0.60±0.03 [d]	1.00±0.06 [b]	1.01±0.09 [b]	1.10±0.10 [b]	0.83±0.03 [c]	1.47±0.07 [a]
210	6-Methyl-5-hepten-2-one	Carotenoids	8.83	2.33	1.28±0.09 [c]	1.49±0.05 [b]	1.19±0.12 [cd]	1.09±0.10 [d]	1.50±0.07 [b]	3.06±0.13 [a]
211	2-Octanone	Lipids	8.92	2.21	0.30±0.02 [c]	0.46±0.04 [b]	0.39±0.04 [bc]	0.40±0.05 [bc]	0.40±0.06 [bc]	1.40±0.11 [a]
212	3-Octen-2-one		10.00	2.33	1.88±0.05 [d]	3.26±0.14 [c]	3.30±0.19 [c]	3.71±0.37 [c]	4.91±0.06 [b]	16.13±1.07 [a]
213	2,2,6-Trimethyl-cyclohexanone	Carotenoids	10.00	2.35	1.33±0.04 [d]	1.77±0.06 [bc]	1.80±0.14 [b]	1.53±0.09 [cd]	1.91±0.05 [b]	4.69±0.27 [a]
214	Isophorone		10.50	2.46	1.91±0.07 [d]	2.63±0.21 [c]	2.36±0.15 [c]	2.30±0.23 [c]	5.57±0.21 [b]	6.47±0.32 [a]
215	(E,E)-3,5-Octadien-2-one		10.58	2.52	2.31±0.21 [d]	4.00±0.25 [c]	3.45±0.40 [cd]	3.40±0.65 [cd]	8.37±0.29 [b]	26.30±1.29 [a]
216	Acetophenone		10.67	2.97	2.25±0.24 [c]	2.62±0.10 [b]	2.87±0.19 [ab]	1.78±0.15 [d]	2.22±0.03 [c]	3.08±0.22 [a]

No.	Compounds	Precursors	RT[a] (min)	RT[b] (s)	Relative contents (μg/L)					
					FL	S1	S2	S3	F	D
217	3-Nonanone		10.92	2.16	0.03±0.00 [c]	0.03±0.00 [c]	0.07±0.01 [b]	0.08±0.01 [b]	0.06±0.00 [b]	0.24±0.02 [a]
218	2-Nonanone	Lipids	11.08	2.17	0.18±0.01 [bc]	0.21±0.02 [b]	0.16±0.00 [bc]	0.14±0.01 [c]	0.19±0.00 [bc]	2.17±0.07 [a]
219	3,5-Octadien-2-one		11.17	2.51	0.28±0.02 [c]	0.52±0.01 [c]	0.47±0.10 [c]	0.39±0.07 [c]	0.99±0.13 [b]	5.85±0.32 [a]
220	(E)-6-Methyl-3,5-heptadien-2-one	Carotenoids	11.33	2.62	-	-	-	-	0.08±0.01	0.37±0.04
221	5-Methyl-2-hexanone		11.75	2.2	0.50±0.02 [d]	0.91±0.06 [c]	0.97±0.06 [c]	1.18±0.09 [b]	1.03±0.02 [c]	4.74±0.11 [a]
222	(R,S)-5-Ethyl-6-methyl-3E-hepten-2-one		12.00	2.25	3.06±0.17 [c]	5.41±0.26 [b]	6.65±0.90 [b]	6.97±1.03 [b]	6.56±0.36 [b]	18.43±1.70 [a]
223	2-Decanone	Lipids	13.00	2.16	0.23±0.01 [d]	1.69±0.06 [c]	2.71±0.22 [b]	3.52±0.48 [a]	1.74±0.09 [c]	1.33±0.08 [c]
224	3,5-Dimethyl-2-octanone		14.33	2.1	0.11±0.00 [f]	0.13±0.00 [e]	0.18±0.01 [c]	0.19±0.00 [b]	0.16±0.00 [d]	0.44±0.01 [a]
225	6-Undecanone		14.42	2.04	0.10±0.01 [c]	0.11±0.00 [c]	0.10±0.01 [c]	0.11±0.01 [c]	0.14±0.00 [b]	0.53±0.04 [a]
226	3-Undecanone	Lipids	14.75	2.14	0.52±0.02 [c]	0.55±0.01 [c]	0.57±0.02 [c]	0.54±0.06 [c]	0.65±0.00 [b]	1.03±0.08 [a]
227	6-Dodecanone		16.17	2.11	-	-	-	-	0.02±0.00	0.10±0.00
**Terpene Ketones (9)**										
228	Menthone		12.42	2.38	0.36±0.03 [c]	0.36±0.04 [c]	0.28±0.04 [d]	0.27±0.03 [d]	0.68±0.03 [a]	0.58±0.02 [b]
229	Isomenthone		12.58	2.45	0.06±0.00 [d]	0.08±0.00 [b]	0.07±0.00 [c]	0.05±0.00 [d]	0.08±0.00 [b]	0.10±0.00 [a]
230	(-)-Carvone		14.08	2.72	0.13±0.00 [d]	0.18±0.01 [c]	0.29±0.05 [a]	0.23±0.03 [bc]	0.24±0.01 [ab]	0.14±0.01 [d]
231	β-Damascenone	Carotenoids;Glycosides	16.42	2.57	-	-	-	-	-	0.20±0.01
232	(Z)-Jasmone	Lipids	16.67	2.83	0.04±0.00 [d]	0.14±0.01 [c]	0.18±0.02 [b]	0.25±0.04 [a]	0.11±0.00 [c]	0.12±0.01 [c]
233	α-Ionone	Carotenoids	17.08	2.48	0.43±0.04 [bc]	0.39±0.02 [c]	0.49±0.05 [bc]	0.45±0.03 [bc]	0.54±0.05 [b]	2.04±0.15 [a]
234	(E)-Geranylacetone	Carotenoids	17.42	2.35	3.99±0.46 [c]	4.47±0.11 [bc]	5.23±0.61 [b]	4.49±0.20 [bc]	4.86±0.07 [b]	13.05±0.70 [a]
235	(E)-β-Ionone	Carotenoids	18.00	2.57	2.21±0.24 [e]	2.46±0.21 [de]	2.97±0.09 [c]	2.71±0.20 [cd]	4.09±0.19 [b]	14.52±0.40 [a]
236	3-Buten-2-one, 4-(2,2,6-trimethyl-7-oxabicyclo[4.1.0]hept-1-yl)-		18.08	2.6	0.21±0.01 [c]	0.22±0.01 [c]	0.28±0.02 [c]	0.31±0.03 [c]	0.47±0.01 [b]	2.72±0.25 [a]
**Others (5)**										
237	Dimethyl sulfide	Maillard reaction	2.33	4.03	2.71±0.12 [c]	7.88±0.46 [c]	4.40±0.52 [c]	9.05±0.81 [c]	31.64±2.26 [b]	73.65±8.15 [a]
238	Hexanoic acid	Lipids	8.75	2.13	-	-	-	-	0.69±0.01	1.60±0.27
239	2-Methoxy-phenol		11.00	2.91	0.29±0.02 [cd]	0.48±0.07 [b]	0.60±0.06 [a]	0.33±0.06 [c]	0.33±0.02 [c]	0.21±0.01 [d]

No.	Compounds	Precursors	RT[a] (min)	RT[b] (s)	Relative contents (μg/L)					
					FL	S1	S2	S3	F	D
240	Theaspirane		15.08	2.26	0.04±0.01 [cd]	0.04±0.00 [cd]	0.03±0.00 [d]	0.04±0.00 [c]	0.08±0.00 [b]	0.35±0.01 [a]
241	Indole	Maillard reaction	15.17	3.47	0.08±0.01 [d]	0.08±0.01 [d]	2.89±0.15 [b]	6.42±0.59 [a]	0.65±0.04 [c]	0.97±0.04 [c]

Note: The relative content of the volatile compounds in sample was expressed as μg/L of ethyl decanoate equivalents; RT[a]: 1st dimensional retention time; RT[b]: 2nd dimensional retention time; "-" The volatile compounds were not detected; In the same row, different letters indicate significant differences (n = 3, $p < 0.05$).

Table S2. Characterization of the volatile compounds in shaken black tea samples during the manufacture based on GC × GC-TOFMS.

No.	Compounds	CAS	RI[a]	RI[b]	Similarity	Reverse
**Alcohols (19)**						
1	2-Methyl-2-propanol	75-65-0	556.14	520	759	895
2	1-Penten-3-ol	616-25-1	708.26	681	928	928
3	1-Pentanol	71-41-0	767.71	764	897	900
4	(Z)-2-Penten-1-ol	1576-95-0	772.66	763	927	927
5	(Z)-3-Hexenol	928-96-1	859.59	858	936	942
6	1-Hexanol	111-27-3	867.99	865	911	911
7	(E)-2-Hexen-1-ol	928-95-0	868.04	862	864	864
8	1-Heptanol	111-70-6	973.72	972	900	900
9	1-Octen-3-ol	3391-86-4	981.71	983	959	959
10	2-Ethyl-1-hexanol	104-76-7	1029.7	1029	948	951
11	Benzyl alcohol	100-51-6	1042.3	1044	953	954
12	(E)-2-Octen-1-ol	18409-17-1	1069.8	1069	762	763
13	1-Methylcycloheptanol	3761-94-2	1115.2		773	802
14	Phenylethyl alcohol	60-12-8	1119.9	1123	961	967
15	(Z)-3-Nonen-1-ol	10340-23-5	1154.1	1152.8	796	843
16	(E,Z)-3,6-Nonadien-1-ol	56805-23-3	1158.5	1156	796	796
17	1-Nonanol	143-08-8	1171.4	1173	848	849
18	1-Decanol	112-30-1	1278.2	1263	812	814
19	Z-2-Dodecenol	69064-36-4	1412.8	1410	861	894
**Terpene alcohols (10)**						
20	(Z)-Linalool oxide (Furanoid)	5989-33-3	1073.8	1070	896	896
21	(E)-Linalool oxide (Furanoid)	34995-77-2	1089.8	1089	919	919
22	Linalool	78-70-6	1101.9	1101	908	908
23	Linalool oxide (Pyranoid)	14049-11-7	1176	1173	865	866
24	Terpinen-4-ol	562-74-3	1189	1179	792	807
25	α-Terpineol	98-55-5	1202.3	1198	822	844
26	(Z)-3,7-Dimethyl-2,6-octadien-1-ol; Nerol	106-25-2	1230.8	1233	900	900
27	Geraniol	106-24-1	1254.7	1254	825	825
28	Nerolidol	142-50-7	1563.6	1565	911	917
29	Cedrol	77-53-2	1632.5	1625	811	815
**Aldehydes (44)**						
30	Acetaldehyde	75-07-0	485.29		909	909
31	2-Propenal	107-02-8	512.28	480	783	861
32	2-Methylpropanal	78-84-2	556.14	548	906	906
33	Butanal	123-72-8	585.96	596	876	879

No.	Compounds	CAS	RI[a]	RI[b]	Similarity	Reverse
34	2-Methylbutanal	96-17-3	670	661	847	850
35	(E)-2-Butenal	123-73-9	683.33	658	886	895
36	Pentanal	110-62-3	717.61	704	883	891
37	(E)-2-Pentenal	1576-87-0	758.9	746	911	911
38	3-Hexenal	6789-80-6	795.23	800	760	793
39	Hexanal	66-25-1	804.35	800	814	814
40	2-Ethyl-3-methyl-butanal	26254-92-2	838.22	833	783	783
41	Furfural	98-01-1	838.86	829	791	754
42	(E)-2-Hexenal	6728-26-3	846.93	844	872	872
43	3-Furaldehyde	498-60-2	855.74	832	845	875
44	2-Hexenal	505-57-7	855.5	853	924	927
45	Heptanal	111-71-7	901.92	902	954	954
46	(Z)-4-Heptenal	6728-31-0	901.98	902	756	882
47	Methional	3268-49-3	910.49	909	875	877
48	(E,E)-2,4-Hexadienal	142-83-6	918.19	914	898	900
49	2,2-Dimethyl-3,4-pentadienal	4058-51-9	945.74		770	834
50	2-Ethylhexanal	123-05-7	957.74	955	760	760
51	(Z)-2-Heptenal	57266-86-1	961.92	958	918	920
52	Benzaldehyde	100-52-7	970.39	962	875	875
53	(E,Z)-2,4-Heptadienal	4313-02-4	1002	1000	883	883
54	Octanal	124-13-0	1005.8	1006	947	951
55	2-Ethyl-2-hexenal	645-62-5	1009.8	1004	809	811
56	(E,E)-2,4-Heptadienal	4313-03-5	1014.1	1012	757	881
57	Benzeneacetaldehyde	122-78-1	1050.4	1043	873	873
58	Aldehydes, 2-(1-pentenyl)-, (E)-	20992-69-2	1057.8	1054	831	836
59	(E)-2-Octenal	2548-87-0	1061.9	1058	951	951
60	2-Methylbenzaldehyde	529-20-4	1074.3	1067	872	904
61	Nonanal	124-19-6	1106.3	1104	887	887
62	(E,E)-2,4-Octadienal	30361-28-5	1115.2	1115	770	777
63	2-Methylenehexanal	1070-66-2	1132.4	1130	788	800
64	(E,Z)-2,6-Nonadienal	557-48-2	1154.3	1158	880	880
65	(E)-2-Nonenal	18829-56-6	1162.9	1156	933	937
66	2,4-dimethyl-Benzaldehyde	15764-16-6	1180.7	1181	871	874
67	Decanal	112-31-2	1206.8	1206	924	924
68	(E,E)-2,4-Nonadienal	5910-87-2	1221.4	1217	939	944
69	2,6,6-Trimethyl-1-cyclohexene-1-acetaldehyde	472-66-2	1264.3	1261	896	897
70	α-Ethylidene-benzeneacetaldehyde	4411-89-6	1279.2	1274	888	893
71	(E,Z)-2,4-Decadienal	25152-83-4	1302.3	1297	774	774

No.	Compounds	CAS	RI[a]	RI[b]	Similarity	Reverse
72	Undecanal	112-44-7	1311.6	1309	937	939
73	(E,E)-2,4-Decadienal	25152-84-5	1326.1	1317	901	902
**Terpene aldehydes (5)**						
74	α-Cyclocitral	432-24-6	1123.8	1122	797	797
75	2,6,6-Trimethyl-1,3-cyclohexadiene-1-carboxaldehyde; Safranal	116-26-7	1207.3	1205	901	903
76	β-Cyclocitral	432-25-7	1226.3	1224	907	909
77	(Z)-3,7-Dimethyl-2,6-octadienal; (Z)-Citral	106-26-3	1245.2	1240	877	884
78	(E)-3,7-Dimethyl-2,6-octadienal; geranial	141-27-5	1273.8	1267	931	931
**Alkanes (17)**						
79	1,1-Dimethyl-cyclopropane	1630-94-0	526.32		903	903
80	n-Hexane	110-54-3	600	600	863	863
81	Octane	111-65-9	799.13	800	886	890
82	Nonane	111-84-2	901.5	900	831	851
83	2,2,4,6,6-Pentamethyl-heptane	13475-82-6	993.36	995	879	879
84	Decane	124-18-5	1001.4	1000	919	922
85	Undecane	1120-21-4	1101.5	1100	938	938
86	5-Methylundecane	1632-70-8	1127.6		825	854
87	3-Methylundecane	1002-43-3	1171.1	1171	884	884
88	Dodecane	112-40-3	1201.7	1200	950	950
89	Tridecane	629-50-5	1301.7	1300	920	929
90	2,6,10-Trimethyl-dodecane	3891-98-3	1335		853	876
91	3-Methyl-tridecane	6418-41-3	1373.1	1377	886	886
92	Tetradecane	629-59-4	1401.9	1400	877	877
93	Pentadecane	629-62-9	1502	1500	891	891
94	3-Methyl-dodecane	17312-57-1	1574.2		835	844
95	Hexadecane	544-76-3	1602.1	1600	901	901
**Alkenes (10)**						
96	(Z)-1,3-Pentadiene	1574-41-0	542.11	510	932	932
97	(E,Z)-1,3,5-Octatriene	40087-61-4	880.6	882	808	824
98	Bicyclo[4.2.0]octa-1,3,5-triene	694-87-1	897.95	895	884	884
99	(Z)-1-Phenylpropene	766-90-5	949.91		827	864
100	α-Methylstyrene	98-83-9	985.93	988	776	798
101	1,4-Hexadiene, 3,3,5-trimethyl-	74753-00-7	1077.5	1078	773	764
102	(E)-4,8-Dimethylnona-1,3,7-triene	19945-61-0	1114.8	1117	886	889
103	(E)-3-Undecene	1002-68-2	1192.9	1191	866	870
104	2,7,7-Trimethyl-bicyclo[2.2.1]hept-2-ene	514-14-7	1240.3		811	816
105	(Z)-3-Tetradecene	41446-67-7	1392.2		913	913
**Terpene alkenes (14)**						

No.	Compounds	CAS	RI[a]	RI[b]	Similarity	Reverse
106	β-Myrcene	123-35-3	993.59	988	949	949
107	α-Phellandrene	99-83-2	1013.6	1007	874	876
108	γ-Terpinene	99-85-4	1021.6	1027	823	823
109	Limonene	138-86-3	1033.7	1032	858	858
110	(E)-β-Ocimene	3779-61-1	1037.8	1038	787	787
111	β-Ocimene	13877-91-3	1049.6	1044	942	942
112	β-Phellandrene	555-10-2	1061.7	1053	863	863
113	Terpinolene	586-62-9	1089.7	1089	873	876
114	β-Elemene	515-13-9	1397.3	1391	809	835
115	Cedrene	11028-42-5	1433.9	1433	775	760
116	(E)-β-Farnesene	18794-84-8	1454.8	1456	865	867
117	α-Farnesene	502-61-4	1507.9	1500	795	798
118	Alloaromadendrene	25246-27-9	1508.1	1496	766	796
119	δ-Cadinene	483-76-1	1530.3	1525	758	814
**Aromatics (30)**						
120	Toluene	108-88-3	772.66	759	896	896
121	Ethylbenzene	100-41-4	863.81	847	958	958
122	1,3-Dimethyl-benzene	108-38-3	872.29	865	962	962
123	p-Xylene	106-42-3	880.25	870	767	786
124	o-Xylene	95-47-6	897.85	894	884	885
125	1-Propylbenzene	103-65-1	957.82	962	918	918
126	1-Ethyl-3-methyl-benzene	620-14-4	965.82	958	913	922
127	(1-Methylethyl)-benzene	98-82-8	985.84	979	793	809
128	1,2,4-Trimethylbenzene	95-63-6	1001.8	1005	935	936
129	o-Cymene	527-84-4	1029.8	1028	919	922
130	1,2,3-Trimethylbenzene	526-73-8	1029.9	1035	866	868
131	Indane	496-11-7	1042	1034	850	863
132	1-Methyl-3-propyl-benzene	1074-43-7	1061.8	1065	801	801
133	2-Ethyl-1,4-dimethyl-benzene	1758-88-9	1089.9		807	862
134	1-Methyl-4-(1-methylethenyl)-benzene	1195-32-0	1093.9	1090	795	831
135	1,2,3,4-Tetramethyl-benzene	488-23-3	1123.8	1122	912	912
136	2,3-Dihydro-4-methyl-1H-indene	824-22-6	1145.7	1151	879	879
137	2-Ethenyl-1,4-dimethyl-benzene	767-58-8	1154.4		849	865
138	Pentylbenzene	538-68-1	1162.8	1159	810	810
139	1,2,3,4-Tetrahydronaphthalene	119-64-2	1171.9	1158	881	881
140	(1,1-Dimethylpropyl)benzene	2049-95-8	1180.2		785	785
141	1-Methyl-4-(1-methyl-2-propenyl)-benzene	97664-18-1	1193.4		843	850
142	1,1-Dimethyl-2,3-dihydro-1H-indene	4912-92-9	1202.4		829	844

No.	Compounds	CAS	RI[a]	RI[b]	Similarity	Reverse
143	1,2,3,4-Tetrahydro-2-methylnaphthalene	3877-19-8	1226.2		752	752
144	1-Methyl-1,2,3,4-tetrahydronaphthalene	1559-81-5	1235.8		809	831
145	1,3,3-Trimethyltricyclo[2.2.1.0(2,6)]heptane	488-97-1	1254.7		888	889
146	2,3-Dihydro-1,3-dimethyl-1H-indene	4175-53-5	1259.6		866	866
147	1,2,3,4-tetrahydro-5-methyl-naphthalene	2809-64-5	1278.7		875	890
148	1-Methyl-naphthalene	90-12-0	1312.3	1299	899	902
149	1,2,3,4-Tetrahydro-1,4-dimethyl-naphthalene	4175-54-6	1331		807	854
**Esters (40)**						
150	Hexanoic acid, methyl ester	106-70-7	925.78	924	756	766
151	Ethyl hexanoate	123-66-0	997.68	1002	913	913
152	Allyl methallyl ether	14289-96-4	997.81		767	792
153	Acetic acid, hexyl ester	142-92-7	1013.7	1012	805	891
154	(Z)-3-Hexen-1-ol, acetate	3681-71-8	1013.8	1009	773	831
155	2-Hexenoic acid, ethyl ester	1552-67-6	1045.8	1043	798	805
156	Formic acid, phenylmethyl ester	104-57-4	1082.3		869	902
157	Heptanoic acid, ethyl ester	106-30-9	1093.7	1093	758	778
158	Methyl benzoate	93-58-3	1098.2	1103	938	938
159	Octanoic acid, methyl ester	111-11-5	1123.6	1125	827	827
160	(E)-Butanoic acid, 3-hexenyl ester	53398-84-8	1141		751	789
161	Acetic acid, 2-ethylhexyl ester	103-09-3	1145.3	1144	921	921
162	Acetic acid, phenylmethyl ester	140-11-4	1167.7	1165	934	934
163	(Z)-3-hexenyl butyrate	16491-36-4	1184.5	1183	833	844
164	Hexyl butyrate	2639-63-6	1188.8	1191	807	862
165	Octanoic acid, ethyl ester	106-32-1	1193.1	1178	755	779
166	Methyl salicylate	119-36-8	1198.1	1193	896	921
167	Nonanoic acid, methyl ester	1731-84-6	1221.1	1225	838	838
168	2-Methyl-pentanoic acid, methyl ester	2177-77-7	1221.2		770	788
169	2-Ethylhexyl acrylate	103-11-7	1225.8	1224	946	946
170	(Z)-3-Hexenyl-α-methylbutyrate	53398-85-9	1235.4	1233	769	781
171	Hexyl 2-methylbutyrate	10032-15-2	1235.3	1239	854	854
172	(Z)-3-Hexenyl isovalerate	35154-45-1	1235.4	1238	808	815
173	(E)-2-Hexenyl isovalerate	68698-59-9	1244.9	1244	877	893
174	Acetic acid, 2-phenylethyl ester	103-45-7	1259.8	1256	774	810
175	Benzoic acid, 2-hydroxy-, ethyl ester	118-61-6	1278.7	1270	859	872
176	Hexanoic acid, pentyl ester	540-07-8	1287.7	1287	852	854
177	Nonanoic acid, ethyl ester	123-29-5	1297.2	1297	841	858
178	n-Butyric acid 2-ethylhexyl ester	25415-84-3	1321	1321	913	918
179	Decanoic acid, methyl ester	110-42-9	1325.8	1328	799	799

No.	Compounds	CAS	RI[a]	RI[b]	Similarity	Reverse
180	Methyl geranate	2349-14-6	1326	1325	809	809
181	Dihydrocarvyl acetate	20777-49-5	1354.6	1344	765	773
182	(Z)-Hexanoic acid, 3-hexenyl ester	31501-11-8	1378.2	1382	898	907
183	Hexanoic acid, hexyl ester	6378-65-0	1382.9	1385	877	877
184	(Z,Z)-3-Hexenoic acid, 3-hexenyl ester	61444-38-0	1383.1	1388	824	863
185	Linalyl isobutyrate	78-35-3	1378.4	1374	755	755
186	Hexanoic acid, 2-hexenyl ester, (E)-	53398-86-0	1387.8	1391	861	861
187	(Z)-3-Hexen-1-ol, benzoate	25152-85-6	1580.7	1580	788	788
188	Benzoic acid, hexyl ester	6789-88-4	1591.7	1595	790	809
189	Hippuric acid, methyl ester	1205-08-9	1597.4		818	975
**Furans (13)**						
190	Furan	110-00-9	526.32		910	910
191	2-Methylfuran	534-22-5	600	599	914	914
192	Tetrahydrofuran	109-99-9	655	623	766	923
193	2-Ethylfuran	3208-16-0	717.61	702	938	938
194	2-Ethyl-5-methyl-furan	1703-52-2	795.23	795	776	761
195	2-Methoxy-furan	25414-22-6	795.23		756	756
196	2-Butyl furan	4466-24-4	893.41	897	906	911
197	2-Pentylfuran	3777-69-3	993.69	993	891	898
198	(E)-2-(2-Pentenyl)furan	70424-14-5	1001.8	1001	918	925
199	2-(2-Propenyl)furan	75135-41-0	1034.2	1030	754	771
200	2,5-Furandicarboxaldehyde	823-82-5	1034.2	1034	818	936
201	2-Hexyl-furan	3777-70-6	1093.7	1096	828	875
202	3-Phenylfuran	13679-41-9	1231.3	1228	902	917
**Ketones (25)**						
203	1-Penten-3-one	1629-58-9	708.26	683	871	875
204	2,3-Hexanedione	3848-24-6	781.47	786	774	870
205	3-Heptanone	106-35-4	885	885	805	838
206	2-Heptanone	110-43-0	889.28	893	933	933
207	6-Methyl-2-heptanone	928-68-7	953.79	956	855	868
208	1-Octen-3-one	4312-99-6	981.82	978	882	887
209	2,5-Octanedione	3214-41-3	985.77	983	823	823
210	6-Methyl-5-hepten-2-one	110-93-0	985.89	985	895	895
211	2-Octanone	111-13-7	993.77	994	845	850
212	3-Octen-2-one	1669-44-9	1041.9	1031	862	866
213	2,2,6-Trimethyl-cyclohexanone	2408-37-9	1041.9	1041	813	836
214	Isophorone	78-59-1	1066	1059	812	819
215	(E,E)-3,5-Octadien-2-one	30086-02-3	1070	1066	908	908

No.	Compounds	CAS	RI[a]	RI[b]	Similarity	Reverse
216	Acetophenone	98-86-2	1074.4	1078	941	941
217	3-Nonanone	925-78-0	1085.7	1087	823	850
218	2-Nonanone	821-55-6	1089.8	1093	802	802
219	3,5-Octadien-2-one	38284-27-4	1094	1095	825	834
220	(E)-6-Methyl-3,5-heptadien-2-one	16647-04-4	1106.6	1107.4	815	852
221	5-Methyl-2-hexanone	110-12-3	1128	1129	776	818
222	(R,S)-5-Ethyl-6-methyl-3E-hepten-2-one	57283-79-1	1141.1	1144	836	836
223	2-Decanone	693-54-9	1193.2	1190	775	762
224	3,5-Dimethyl-2-octanone	19781-14-7	1268.7		804	810
225	6-Undecanone	927-49-1	1273.4	1274	885	893
226	3-Undecanone	2216-87-7	1292.5	1283	801	801
227	6-Dodecanone	6064-27-3	1373.4	1375	793	794
**Terpene Ketones (9)**						
228	Menthone	89-80-5	1162.9	1153	844	848
229	Isomenthone	1196-31-2	1171.7	1163	761	783
230	(-)-Carvone	6485-40-1	1255	1254	782	789
231	β-Damascenone	23696-85-7	1388.2	1390	783	802
232	(Z)-Jasmone	488-10-8	1403	1398	763	800
233	α-Ionone	127-41-3	1428.9	1427	872	872
234	(E)-Geranylacetone	3796-70-1	1449.8	1452	935	952
235	(E)-β-Ionone	79-77-6	1486.9	1485	942	942
236	3-Buten-2-one, 4-(2,2,6-trimethyl-7-oxabicyclo[4.1.0]hept-1-yl)-	23267-57-4	1492.2	1484	925	925
**Others (5)**						
237	Dimethyl sulfide	75-18-3	526.32	515	958	958
238	Hexanoic acid	142-62-1	981.73	981	810	816
239	2-Methoxy-phenol	90-05-1	1090.3	1090	775	760
240	Theaspirane	36431-72-8	1311.7	1302	836	837
241	Indole	120-72-9	1320.8	1300	921	921

Note: RI[a]: The retention index (RI) was calculated by the n-alkane standards (C_3–C_{40}); RI[b]: The RI reported in references (http://webbook.nist.gov/chemistry/ and http://www.flavornet.org/flavornet.html/).

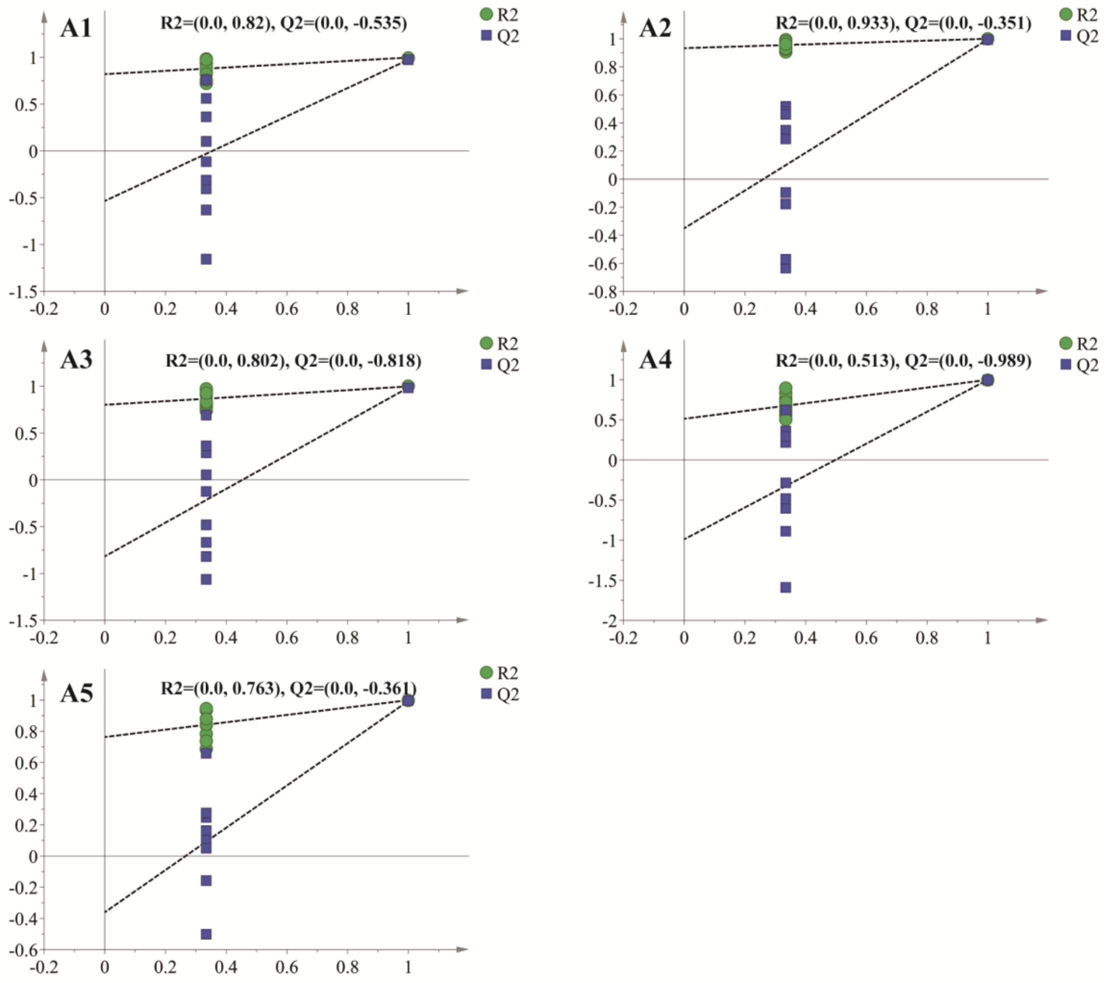

Figure S1. (A1) permutation plot for the OPLS-DA model (FL *vs.* S1); (A2) permutation plot for the OPLS-DA model (S1 *vs.* S2); (A3) permutation plot for the OPLS-DA model (S3 *vs.* S2); (A4) permutation plot for the OPLS-DA model (S3 *vs.* F); (A5) permutation plot for the OPLS-DA model (F *vs.* D).
